# *FURIN*, *IFNL4,* and *TLR2* gene polymorphisms in relation to COVID-19 severity: a case–control study in Egyptian patients

**DOI:** 10.1007/s15010-024-02266-1

**Published:** 2024-05-04

**Authors:** Gamalat A. Elgedawy, Naglaa S. Elabd, Radwa H. Salem, Samah M. Awad, Amany A. Amer, Mohammad M. Torayah, Amal A. El-Koa, Mai Abozeid, Belal A. Montaser, Hind S. Aboshabaan, Mervat Abdelkreem, Marwa L. Helal

**Affiliations:** 1 Department of Clinical Biochemistry and Molecular Diagnostics, National Liver Institute, Menoufia University, Shebin El-Kom, Menoufia, Egypt; 2 Faculty of Medicine, Department of Tropical Medicine, Menoufia University, Shebin El-Kom, Menoufia, 32511 Egypt; 3 Department of Clinical Microbiology and Immunology, National Liver Institute, Menoufia University, Shebin El-Kom, Menoufia, Egypt; 4 Faculty of Medicine, Department of Critical Care Medicine, Menoufia University, Shebin El-Kom, Egypt; 5 Faculty of Medicine, Department of Chest Diseases and Tuberculosis, Menoufia University, Shebin El‑Kom, Menoufia, Egypt; 6 Department of Hepatology and Gastroenterology, National Liver Institute, Menoufia University, Shebin El-Kom, Menoufia, 32511 Egypt; 7 Faculty of Medicine, Department of Clinical Pathology, Menoufia University, Shebin El-Kom, Menoufia, Egypt; 8 Ph.D. of Biochemistry, National Liver Institute, Menoufia University, Shebin El-Kom, Menoufia, Egypt

**Keywords:** *FURIN*, *IFNL4*, *TLR2*, Gene variants, Genotypes, Alleles

## Abstract

**Background and Aim:**

A wide range of clinical manifestations and outcomes, including liver injury, have been reported in COVID-19 patients. We investigated the association of three substantial gene polymorphisms (*FURIN*, *IFNL4*, and *TLR2*) with COVID-19 disease susceptibility and severity to help predict prognosis.

**Methods:**

150 adult COVID-19-assured cases were categorized as follows: 78 patients with a non-severe presentation, 39 patients with severe disease, and 33 critically ill patients. In addition, 74 healthy controls were included. Clinical and laboratory evaluations were carried out, including complete and differential blood counts, D-dimer, lactate dehydrogenase (LDH), C-reactive protein (CRP), procalcitonin, ferritin, interleukin-6 (Il-6), and liver and kidney functions. *FURIN* (rs6226), *IFNL4* (rs12979860), and *TLR2* (rs3804099) genotyping allelic discrimination assays were conducted using real-time PCR.

**Results:**

The *FURIN*, *IFNL4*, and *TLR2* genotypes and their alleles differed significantly between COVID-19 patients and controls, as well as between patients with severe or critical illness and those with a non-severe presentation. According to a multivariable regression analysis, *FURIN* (C/T + T/T) and *TLR2* (T/C + C/C) mutants were associated with COVID-19 susceptibility, with odds ratios of 3.293 and 2.839, respectively. *FURIN* C/C and *IFNL4* T/T mutants were significantly linked to severe and critical illnesses. Multivariate regression analysis showed that *FURIN* (G/C + C/C) genotypes and *IFNL4* T/T homozygosity were independent risk factors associated with increased mortality.

**Conclusion:**

*FURIN*, *IFNL4*, and *TLR2* gene variants are associated with the risk of COVID-19 occurrence as well as increased severity and poor outcomes in Egyptian patients.

**Supplementary Information:**

The online version contains supplementary material available at 10.1007/s15010-024-02266-1.

## Introduction

Coronavirus disease 2019 (COVID-19) is a contagious pneumonia-like illness caused by the coronavirus 2 severe acute respiratory syndrome (SARS-CoV-2). The first outbreak was identified in December 2019 in Wuhan, China, and it rapidly spread throughout the world, causing the COVID-19 pandemic [1]. The clinical manifestations vary widely, with fatigue, fever, and cough being the most prevalent. Additionally, some patients presented with a sore throat, congestion, rhinorrhea, and gastrointestinal symptoms including vomiting and diarrhea [2]. Liver injury was reported in most studies to be correlated with COVID-19 severity, although liver failure is unusual [3]. COVID-19 has also been linked to immune dysfunction, acute respiratory distress syndrome, and multiorgan failure. The mechanisms included abnormal renin-angiotensin system activity, cytokine storm, oxidative stress, neutrophil stimulation, and vitamin D receptor gene expression, as well as enhanced coagulopathies [4].

It has been thought that host genetic variations, particularly those associated with immune responses, determine the patients’ susceptibility and COVID‐19 disease severity [5]. The *FURIN* gene encodes a membrane-bound protease of the subtilisin-like proprotein convertase family. It is utilized by various pathogens to process their envelope proteins [6]. The spike glycoprotein of the COVID‐19 virus has a *FURIN* cleavage site that facilitates viral transmission into or from the host cell. The *FURIN* rs6226 is one of the most prevalent *FURIN* variants in African and Middle Eastern populations, but not in European populations [7]. Lambda interferons (IFNLs) include IFNL1/interleukin-29 (IL-29), IFNL2/IL‐28A, IFNL3/IL‐28B, and IFNL4, all of which are critical for a balanced antiviral response in the respiratory tract for optimal protection against infection and minimizing associated damage [8]. The single nucleotide polymorphism (SNP) rs12979860 is commonly considered an *‘IFNL3/IL28B’* variant, but it’s located in intron 1 of *IFNL4* and, therefore, is properly termed *IFNL4* rs12979860 [9]. Recent studies found that rs12979860 can predispose to COVID-19 infection and indicated a strong relationship between rs12979860 SNPs and the severity of infection [10, 11]. Toll-like receptor 2 (TLR2) could recognize pathogen-associated molecular patterns in addition to responding to pathogens by inducing an acquired immune response. *TLR2* rs3804099 is a common genetic mutation site correlated with viral liver disease [12]. *TLR2* expression was found to increase with COVID-19 severity [13]. *TLR2* rs3804099 has been associated with pulmonary infection [14]. However, data about its role in COVID-19 or associated liver injury is lacking.

To our knowledge, no study in Egypt has investigated the impact of *FURIN* rs6226, *IFNL4* rs12979860, or *TLR2* rs3804099 variants in COVID-19 patients. Therefore, the present study aimed to evaluate the association of these three substantial gene polymorphisms with COVID-19 disease susceptibility and severity in Egyptian patients.

## Materials and methods

### Study design and participants

This case–control study was conducted on 150 adult COVID-19-assured cases recruited from the Departments of Tropical Medicine, Chest, and Intensive Care Unit of Menoufia University Hospital in collaboration with the National Liver Institute Hospital from September 2022 to February 2023. Patients 18 years of age or older who had COVID-19 confirmed by polymerase chain reaction (PCR) from nasopharyngeal and oropharyngeal samples were included. Exclusion criteria included patients with pre-existing cardiac, hepatic, renal, chest, or coagulation disorders. In addition to COVID-19-vaccinated participants and those with a previous history of COVID-19, all patients who declined to participate in the study, skipped the study, or lacked the necessary data were not included. Furthermore, 74 healthy participants were included in the healthy control (HC) group. HC were selected from healthcare providers who appeared healthy and showed no signs of COVID-19 infection based on common clinical criteria and laboratory testing (scheduled screening for health care workers by detection for both S and A antigens). The Epi-Info website was utilized for calculating the sample size (https://www.cdc.gov/Epi-Info/unmatched-case-control).

### Clinical assessment of studied patients

Throughout the research period, patients who presented to the COVID-19 isolation unit at the Faculty of Medicine, Menoufia University Hospital, with a clinical suspicion of the virus were evaluated. Confirmed cases were categorized as non-severe, severe, or critical after evaluations by clinical, laboratory, and radiological methods. In cases deemed non-severe, a prescription for outpatient treatment was given, and additional follow-up was done via phone or at the COVID-19 outpatient clinic. COVID-19 patients exhibiting severe or critical symptoms were admitted to the COVID-19 isolation ward or intensive care unit (ICU), where initial clinical, laboratory, and radiological data were documented. In addition, a daily assessment of the illness’s progression and the patient’s response to treatment were noted and assessed. Patients were monitored until their death or discharge from the hospital.

COVID-19 patients were classified based on CT presentations using the COVID-19 Reporting and Data System (CO-RAD) radiological score. The findings of CO-RADS 1 are either normal or non-infectious; CO-RADS 2 is idealistic for other infectious causes but not COVID-19 pneumonia; and CO-RADS 3 is comparable to COVID-19 pneumonia in addition to other illnesses. CO-RADS 4 and 5 are highly compatible with COVID-19 pneumonia, but with atypical features as well in CO-RADS 4 [15]. According to the World Health Organization (WHO) guidelines [16], patients were divided into three categories as follows: **The non-severe COVID-19 group** included 78 patients not meeting either critical or severe COVID-19 criteria. **The severe COVID-19 group** included 39 patients with any of the following criteria: on room air, oxygen saturation < 90%, or evidence of severe respiratory distress (respiratory rate > 30 breaths per minute, difficulty finishing the entire sentence, or using accessory muscles of respiration), in addition to signs of pneumonia. **The critically ill COVID-19 group** included 33 patients who met the criteria for acute respiratory distress syndrome, sepsis, septic shock, or conditions requiring life-sustaining therapeutic strategies.

### Laboratory investigations

10 mL of blood samples were collected from all participants after overnight fasting and aliquoted into ethylenediaminetetraacetic acid (EDTA)-containing tubes, sodium citrate-containing tubes, and plain tubes for SNPs, CBC analysis, coagulation parameters, and biochemical investigations, respectively. After centrifugation at high speed for 10 min, sera were analyzed for blood chemistry (LDH, CRP, blood sugar, liver and kidney function tests, and lipid profile) using a fully automated chemistry analyzer, SYNCHRON CX9ALX Beckman Coulter (CA, USA), which was also utilized to estimate D-dimer. CBC was assayed by a semi-automated Sysmex analyzer (Siemens, Germany), INR/PT (The Sysmex CA-600 Systems, Siemens, Germany), and serum ferritin (Abbott chemiluminescence instrument, Architect, USA).

### IL-6 assay

The IL-6 Human ELISA Kit (Invitrogen, USA) was utilized and then measured by a Multiskan Sky Microplate Spectrophotometer (Thermo Fisher Scientific, USA).

### For health care workers (control group)

During scheduled screening, five mL of the blood were delivered to a vacutainer plain test tube. Serum was separated by centrifugation at 3000 rpm for 10 min and was used for detection of the specific anti-COVID-19 antibody by chemiluminescence immunoassay seronegative for IgG for S or N using Cobas 6000 (Roche, Germany).

### COVID-19 RNA analysis

RNA extraction of nasopharyngeal swab specimens was performed using the RNeasy Mini Kit (Qiagen, Hilden, Germany). Then, the cDNA first strand was prepared using the cDNA synthesis kit (Invitrogen) and specific primers using the Proflex cycler (USA). The diagnosis of COVID-19 was confirmed by a positive fluorescent signal and RT-PCR detection of cDNA. The signal reflects a positive test result.

### SNP analyses of *FURIN*, *IFNL4*, and *TLR2* genes

Genotyping of *FURIN* (rs6226), *IFN4* (rs12979860), and *TLR2* (rs3804099) genes was performed using allelic discrimination techniques that detect the variants of the gene. Unknown samples were classified as homozygotes (samples with only allele 1 or allele 2) and heterozygotes (samples with both allele 1 and allele 2). Genomic DNA extraction was conducted using a spin column method (Thermo Scientific, Lithuania, GeneJET whole blood genomic DNA purification mini kit). DNA concentration was measured using NanoDrop spectrophotometer (Thermofisher Scientific, USA). PCR was performed on a real-time PCR system (7500 Fast, Applied Biosystems, USA) using the TaqMan SNP genotyping assay: primers and probe (40  ×) (Thermo Fisher Scientific, MA, USA) and genotyping qPCR Master Mix (2 ×). For the amplification reaction, the master mix (total volume: 20 µL) consisted of 10 µL of genotyping qPCR master mix, 0.5 µL of genotyping assay, and 3.5 µL of DNAse-free water; then, 6 µL of extracted genomic DNA template was added. 6 µL of DNAse-free water was then added as a negative control reaction. The cycling parameters were set as follows: holding stage (pre-PCR): 60 °C for 1 min, initial denaturation step: 95 °C for 10 min, cycling stage: denaturation step: 95 °C for 1 min repeated for 35 cycles, annealing and extension: 60 °C for 1 min, and post-PCR (holding stage): 60 °C for 1 min. The TaqMan assays were predesigned (for *FURIN* rs6226: C_11947693_10, for *INF4* rs12979860: C_7820462_10, and for TLR2 rs3804099: C_22274563_10).

### Statistical analysis

Data were analyzed using IBM SPSS package version 20.0 (Armonk, NY: IBM Corp.). Categorical information was delineated as numbers and percentages. The Chi-square (χ2) test was used for comparing between two groups. Alternatively, the Fisher Exact correction test was applied when > 20% of the cells had an expected count < 5, and the Monte Carlo correction test was applied when > 20% of the cells had an anticipated count of < 5. The Kolmogorov–Smirnov test was applied to test for normality for continuous data. Quantitative data were expressed as range (minimum and maximum), mean, standard deviation, and median. For non-normally distributed quantitative variables, the Mann–Whitney test was applied for comparing between two groups, whereas the Kruskal–Wallis test was used to compare between more than two groups, and then the Post-Hoc test (Dunn’s multiple comparisons test) was utilized for pairwise comparison. The population of the studied sample was investigated in order to determine its equilibrium using the Hardy–Weinberg equation. The 95% confidence interval (CI) and odds ratio (OR) were calculated to assess the effects of alleles and genotypes. For additional analysis, OR was done in various genetic models (dominant, recessive, and additive). Regression analysis was performed to detect the independent factor (s) for COVID-19 susceptibility, severity, and mortality. A *P* value of < 0.05 was considered statistically significant.

## Results

The biochemical, hematological, and demographic characteristics of COVID-19 patients and control subjects were summarized in Table 1**.** There was no significant difference in sex between COVID-19 patients and controls; however, COVID-19 patients were significantly older than controls. A highly significant difference was found in cases versus controls regarding ALT, CBC, CRP, urea, creatinine, ferritin, D-dimer, procalcitonin, albumin, total bilirubin, LDH, PT, INR, and IL6. All were increased in COVID-19 patients except for HB, platelets, and lymphocytes, which were decreased.

**Table 1:** Comparison between COVID-19 patients and control groups according to demographic and laboratory data

	Reference range	Total patients (*n* = 150)	Controls (*n* = 74)	Test of Sig	*p*
*Sex*					
Male		84 (56%)	38 (51.4%)	χ^2^ = 0.432	0.511
Female		66 (44%)	36 (48.6%)		
*Age* (years)					
Mean ± SD		57.3 ± 16.3	39.5 ± 5.5	U = 1917.0*	< 0.001*
Median (Min.–Max.)		58.5 (25–88)	39 (25–54)		
*Hb*	(12–16 g/dL)				
Mean ± SD		11 ± 1.6	12.5 ± 0.7	U = 2611.50*	< 0.001*
Median (Min.–Max.)		11 (7.5–14)	12.5 (11.5–14)		
*WBCs*	(4.4–11 × 1000/µL)				
Mean ± SD		9.4 ± 5.9	4.6 ± 0.6	U = 2235.00*	< 0.001*
Median (Min.–Max.)		9 (3.8–32)	4.3 (4–6)		
*Lymphocytes*	(20–40%)				
Mean ± SD		13.9 ± 4.1	32.4 ± 4.8	U = 13.50*	< 0.001*
Median (Min.–Max.)		13 (5–23)	33 (20–40)		
*Segmented*	(47–55%)				
Mean ± SD		79.3 ± 8	51.5 ± 5	U = 222.0*	< 0.001*
Median (Min.–Max.)		80 (38–90)	52 (42–60)		
*Platelet count*	(150–450 × 1000/µL)				
Mean ± SD		253.8 ± 827	364.8 ± 51.7	U = 1627.50*	< 0.001*
Median (Min.–Max.)		230 (129–450)	378 (233–432)		
*CRP*	(8–10 mg/L)				
Mean ± SD		74.7 ± 54	3.7 ± 1.4	U = 0.0*	< 0.001*
Median (Min.–Max.)		48 (12–196)	4 (1–6)		
*Serum creatinine*	(0.7–1.3 mg/dL)				
Mean ± SD		1.1 ± 0.25	0.8 ± 0.15	U = 975.0*	< 0.001*
Median (Min.–Max.)		1 (0.8–2.1)	0.8 (0.3–0.9)		
*Blood urea*	(15–40 mg/dL)				
Mean ± SD		44.1 ± 17.8	21.6 ± 4.6	U = 294.0*	< 0.001*
Median (Min.–Max.)		40 (25–102)	20 (12–31)		
*ALT*	(4–36 IU/L)				
Mean ± SD		43.1 ± 37	25.3 ± 7.1	U = 2959.50*	< 0.001*
Median (Min.–Max.)		35 (18–250)	25 (17–36)		
*Serum ferritin*	(12–300 ng/mL)				
Mean ± SD		681.2 ± 408.6	277 ± 39.9	U = 663.00*	< 0.001*
Median (Min.–Max.)		500 (200–2000)	295.5 (190–335)		
*LDH*	(140–280 IU/L)				
Mean ± SD		649 ± 378.1	207.8 ± 45.8	U = 0.000*	< 0.001*
Median (Min.–Max.)		600 (280–1800)	199 (105–276)		
*D-Dimer*	(< 0.5 mg/L)				
Mean ± SD		1 ± 0.82	0.23 ± 0.08	U = 1378.50*	< 0.001*
Median (Min.–Max.)		1 (0.10–3.5)	0.20 (0.10–0.40)		
*Prolcalcitonin*	(< 0.1 ng/mL)				
Mean ± SD		0.41 ± 0.45	0.008 ± 0.01	U = 247.50*	< 0.001*
Median (Min.–Max.)		0.23 (0.01–1.50)	0 (0–0.04)		
*PT*	(11–13.5 s)				
Mean ± SD		13.2 ± 1.1	12.6 ± 0.69	U = 3865.50*	< 0.001*
Median (Min.–Max.)		13 (11–15)	13 (11–13.5)		
*INR*	(< 1.1)				
Mean ± SD		1 ± 0.07	0.997 ± 0.005	U = 3138.00*	< 0.001*
Median (Min.–Max.)		1 (0.8–1.3)	1 (0.99–1)		
*IL-6*	(< 10 pg/mL)				
Mean ± SD		174.5 ± 84.8	11.1 ± 4.3	U = 22.50*	< 0.001*
Median (Min.–Max.)		202.5 (20–350)	11 (4–22)		

*Hb* Hemoglobin concentration, *WBCs* White blood cells, *CRP* C reactive protein, *ALT* Alanine transaminase, *LDH* Lactate Dehydrogenase, *PT* Prothrombin time, *INR* International normalized ratio, *IL-6* Interleukin-6, *SD* Standard deviation, *U* Mann Whitney test, *χ*^2^ Chi square test, *p* *p* value, *Statistically significant at *p* < 0.05

There was a significant variation in clinical symptoms and signs, severity score (CO-RADS), and outcome between the three stages of COVID-19 patients, with 57.6% and 28.2% dying in the critical and severe stages, respectively (Table 2). A significant difference was found among the four studied groups in terms of sex and age. Laboratory findings were more evident in the severe and critical groups than in the non-severe group. The total leucocyte count, ALT, INR, urea, and creatinine, as well as inflammatory indices (CRP, LDH, ferritin, and prolcalcitonin), showed the highest values in severe and critically ill patients. IL-6 levels were rising with increasing COVID-19 severity and recorded the lowest levels in the control group (Table 3).

**Table 2:** Comparison between the three studied patients groups according to clinical assessment

	Non-severe (*n* = 78)	Severe (*n* = 39)	Critical (*n* = 33)	χ^2^	*p*
Fever	54 (69.2%)	39 (100.0%)	33 (100.0%)	26.374*	< 0.001*
Cough	66 (84.6%)	39 (100.0%)	33 (100.0%)	11.609*	^MC^*p* = 0.002*
Sputum	21 (26.9%)	30 (76.9%)	24 (72.7%)	34.741*	< 0.001*
Dyspnea	36 (46.2%)	39 (100.0%)	33 (100.0%)	53.846*	< 0.001*
Hemoptysis	0 (0.0%)	3 (7.7%)	0 (0.0%)	5.882*	^MC^*p* = 0.028*
Cyanosis	0 (0.0%)	12 (30.8%)	6 (18.2%)	27.773*	^MC^*p* < 0.001*
Myalgia	24 (30.8%)	12 (30.8%)	6 (18.2%)	2.023	0.364
Bone ache	36 (46.2%)	21 (53.8%)	6 (18.2%)	10.484*	0.005*
Anosmia	51 (65.4%)	18 (46.2%)	6 (18.2%)	20.979*	< 0.001*
Loss of taste	33 (42.3%)	6 (15.4%)	3 (9.1%)	16.852*	< 0.001*
Vomiting	21 (26.9%)	3 (7.7%)	3 (9.1%)	8.790*	0.012*
Diarrhea	48 (61.5%)	15 (38.5%)	3 (9.1%)	26.544*	< 0.001*
Conjunctivitis	15 (19.2%)	18 (46.2%)	21 (63.6%)	22.205*	< 0.001*
Chills	6 (7.7%)	6 (15.4%)	6 (18.2%)	3.223	^MC^*p* = 0.212
Runny nose	18 (23.1%)	0 (0.0%)	0 (0.0%)	19.986*	^MC^*p* < 0.001*
History of DM	21 (26.9%)	27 (69.2%)	27 (81.8%)	35.748*	< 0.001*
History of HTN	18 (23.1%)	27 (69.2%)	24 (72.7%)	34.463*	< 0.001*
*CO-RADS*					
0	8 (10.3%)	0 (0.0%)	0 (0.0%)	63.977*	^MC^*p* < 0.001*
3	7 (9.0%)	0 (0.0%)	0 (0.0%)		
4	45 (57.7%)	6 (15.4%)	3 (9.1%)		
5	18 (23.1%)	33 (84.6%)	30 (90.9%)		
*Outcome*					
Alive	78 (100.0%)	28 (71.8%)	14 (42.4%)	50.262*	< 0.001*
Died	0 (0.0%)	11 (28.2%)	19 (57.6%)		

*DM* Diabetes mellitus, *HTN* Hypertension, *χ*^2^ Chi square test, *MC* Monte Carlo, *p* *p* value, *Statistically significant at *p* < 0.05

**Table 3:** Comparison between the four studied groups according to demographic and laboratory data

	Non-severe (*n* = 78)	Severe (*n* = 39)	Critical (*n* = 33)	Control (*n* = 74)	Test of Sig	*p*
*Sex*						
Male	48 (61.5%)	12 (30.8%)	24 (72.7%)	38 (51.4%)	χ^2^ = 15.130*	0.002*
Female	30 (38.5%)	27 (69.2%)	9 (27.3%)	36 (48.6%)		
*Age* (*years*)						
Mean ± SD	51.2 ± 16.7	58.5 ± 10.1	70.4 ± 13.5	39.5 ± 5.5	H = 96.055*	< 0.001*
Median (Min.–Max.)	46^c^ (25–80)	57^b^ (42–80)	75^a^ (48–88)	39^d^ (25–54)		
*Hb* (*12–16 *g/dL)						
Mean ± SD	11.2 ± 1.5	11 ± 1.6	10.6 ± 1.8	12.5 ± 0.7	H = 44.417*	< 0.001*
Median (Min.–Max.)	11^b^ (8.5–14)	11^b^ (8.5–14)	10.5^b^ (7.5–14)	12.5^a^ (11.5–14)		
*WBCs* (*4.4–11 × 1000*/µL)						
Mean ± SD	8.8 ± 4.5	9.2 ± 3	11.3 ± 9.9	4.6 ± 0.6	H = 56.640*	< 0.001*
Median (Min.–Max.)	8.8^a^ (3.8–25)	10^a^ (3.9–15)	5^a^ (3.8–32)	4.3^b^ (4–6)		
*Lymphocytes *(*20–40*%*)*						
Mean ± SD	14.9 ± 4	13.6 ± 4	12.1 ± 4.2	32.4 ± 4.8	H = 152.837*	< 0.001*
Median (Min.–Max.)	15^b^ (7–23)	13^bc^ (5–22)	12^c^ (7–20)	33^a^ (20–40)		
*Segmented* (*47–55*%)						
Mean ± SD	77.4 ± 9.2	80.6 ± 4.5	82.2 ± 7.1	51.5 ± 5	H = 143.996*	< 0.001*
Median (Min.–Max.)	78^b^ (38–90)	80^ab^ (70–90)	83^a^ (70–90)	52^c^ (42–60)		
*Platelet count (150–450 × 1000/µL)*						
Mean ± SD	251.3 ± 75.4	231.7 ± 66.9	285.8 ± 103.4	364.8 ± 51.7	H = 79.990*	< 0.001*
Median (Min.–Max.)	231.5^bc^(129–430)	230^c^ (145–377)	297^b^ (140–450)	378^a^ (233–432)		
*CRP (8–10 mg/L)*						
Mean ± SD	49.2 ± 37.3	81.5 ± 49.9	127.1 ± 52.8	3.7 ± 1.4	H = 175.125*	< 0.001*
Median (Min.–Max.)	45.5^c^ (12–169)	48^b^ (24–196)	96^a^ (48–196)	4^d^ (1–6)		
*Serum Creatinine (0.7–1.3 mg/dL)*						
Mean ± SD	1.4 ± 0.24	1.4 ± 0.18	1.25 ± 0.29	0.8 ± 0.15	H = 117.718*	< 0.001*
Median (Min.–Max.)	1^b^ (0.8–2.1)	1^b^ (0.8–1.5)	1.20^a^ (1–1.9)	0.8^c^ (0.3–0.9)		
*Blood urea (15–40 mg/dL)*						
Mean ± SD	40.2 ± 13.9	43.2 ± 14.6	54.7 ± 24.6	21.7 ± 4.6	H = 139.690*	< 0.001*
Median (Min.–Max.)	39^b^ (25–100)	40^ab^ (25–86)	45^a^ (25–102)	20^c^ (12–31)		
*ALT (4–36 IU/L)*						
Mean ± SD	36.2 ± 22.2	42.9 ± 26	59.6 ± 63.2	25.3 ± 7.1	H = 40.061*	< 0.001*
Median (Min.–Max.)	28^b^ (18–120)	35^ab^ (18–110)	40^a^ (20–250)	25^c^ (17–36)		
*Serum ferritin (12–300 ng/mL)*						
Mean ± SD	469.4 ± 262.9	765.4 ± 294.3	1082.3 ± 473.6	277 ± 39.9	H = 147.347*	< 0.001*
Median (Min.–Max.)	352.5^b^ (300–1500)	700^a^ (200–1200)	1000^a^ (500–2000)	295.5^c^ (190–335)		
*LDH (140–280 IU/L)*						
Mean ± SD	433.1 ± 204.3	741.5 ± 247.1	1050 ± 450.5	207.8 ± 45.8	176.918*	< 0.001*
Median (Min.–Max.)	340^b^(280–1000)	750^a^(290–1200)	1000^a^(400–1800)	199^c^ (105–276)		
*D-Dimer (< 0.5 mg/L)*						
Mean ± SD	0.6 ± 0.6	1.3 ± 0.8	1.6 ± 0.7	0.23 ± 0.08	131.447*	< 0.001*
Median (Min.–Max.)	0.4^b^ (0.1–3)	1^a^ (0.5–3.5)	1.5^a^ (0.6–3.5)	0.2^c^ (0.1–0.4)		
*Prolcalcitonin (< 0.1 ng/mL)*						
Mean ± SD	0.3 ± 0.4	0.4 ± 0.3	0.6 ± 0.5	0 ± 0	145.220*	< 0.001*
Median (Min.–Max.)	0.2^b^ (0–1.5)	0.4^ab^ (0–1.1)	0.4^a^ (0.1–1.5)	0^c^ (0–0)		
*PT (11–13.5 s)*						
Mean ± SD	12.9 ± 0.9	13.2 ± 1.3	13.9 ± 0.7	12.6 ± 0.7	40.413*	< 0.001*
Median (Min.–Max.)	13^bc^ (12–15)	13^b^ (11–15)	14^a^ (13–15)	13^c^ (11–13.5)		
INR (< 1.1)						
Mean ± SD	1.02 ± 0.08	0.97 ± 0.08	1.01 ± 0.03	1 ± 0	42.804*	< 0.001*
Median (Min.–Max.)	1^a^ (0.9–1.3)	1^c^ (0.8–1)	1^a^ (1–1.1)	1^b^ (1–1)		
*IL-6 (< 10 pg/mL)*						
Mean ± SD	138.5 ± 82.1	213.2 ± 92.2	213.6 ± 25.4	11.1 ± 4.3	161.490*	< 0.001*
Median (Min.vMax.)	147.5^b^ (20–320)	240^a^ (20–350)	215^a^ (180–260)	11^c^ (4–22)		

*Hb* Hemoglobin concentration, *WBCs* White blood cells, *CRP* C reactive protein, *ALT* Alanine transaminase, *LDH* Lactate Dehydrogenase, *PT* Prothrombin time, *INR* International normalized ratio, *SD* Standard deviation, *χ*^2^ Chi square test, *H* H for Kruskal Wallis test, Pairwise comparison between 2 groups was done using Post Hoc Test (Dunn’s for multiple comparisons test), *p* *p* value, *Statistically significant at *p* < 0.05, means/medians with common letters are not significant (i.e. means/medians with different letters are significant)

Hardy–Weinberg equilibrium for *FURIN* rs6226, *IFNL4* rs12979860, and *TLR2* rs3804099 genotype distribution showed non-significant differences in patients and controls as well as in non-severe, severe, and critical patients’ groups (Tables 4, 5). The genotypes, alleles, and genetic models (dominant and recessive), differed significantly between COVID-19 patients compared to controls (Table 4). Also, a significant difference was found when patients with severe or critical illnesses were compared to those with non-severe presentations. However, there was no variation between non-severe and control groups as well as between severe and critical groups (Table 5).

**Table 4:** Comparison between COVID-19 patients and control groups according to different SNPs

	Total patients (*n* = 150)	Control (*n* = 74)	χ^2^	*p*
*FURIN* rs6226				
G/G®	40 (26.7%)	37 (50%)	13.627*	0.001*
G/C	63 (42%)	26 (35.1%)		
C/C	47 (31.3%)	11 (14.9%)		
^HW^p	0.053	0.088		
*Allele*				
G®	143 (47.7%)	100 (67.6%)	15.814*	< 0.001*
C	157 (52.3%)	48 (32.4%)		
*Dominant genetic model*				
G/G	40 (26.7%)	37 (50.0%)	11.960*	0.001*
G/C + C/C	110 (73.3%)	37 (50.0%)		
*Recessive genetic model*				
G/G + G/C	103 (68.7%)	63 (85.1%)	7.004*	0.008*
C/C	47 (31.3%)	11 (14.9%)		
*IFNL4 rs12979860*				
C/C®	54 (36%)	39 (52.7%)	7.338*	0.026*
C/T	62 (41.3%)	27 (36.5%)		
T/T	34 (22.7%)	8 (10.8%)		
^HW^p	0.052	0.323		
*Allele*				
C®	170 (56.7%)	105 (70.9%)	8.525*	0.004*
T	130 (43.3%)	43 (29.1%)		
*Dominant genetic model*				
C/C	54 (36.0%)	39 (52.7%)	5.694*	0.017*
C/T + T/T	96 (64.0%)	35 (47.3%)		
*Recessive genetic model*				
C/C + C/T	116 (77.3%)	66 (89.2%)	4.572*	0.032*
T/T	34 (22.7%)	8 (10.8%)		
*TLR2 rs3804099*				
T/T®	35 (23.3%)	36 (48.6%)	16.291*	< 0.001*
T/C	64 (42.7%)	26 (35.1%)		
C/C	51 (34%)	12 (16.2%)		
^HW^p	0.094	0.065		
*Allele*				
T®	134 (44.7%)	98 (66.2%)	18.433*	< 0.001*
C	166 (55.3%)	50 (33.8%)		
*Dominant genetic model*				
T/T	35 (23.3%)	36 (48.6%)	14.669*	< 0.001*
T/C + C/C	115 (76.7%)	38 (51.4%)		
*Recessive genetic model*				
T/T + T/C	99 (66.0%)	62 (83.8%)	7.753*	0.005*
C/C	51 (34.0%)	12 (16.2%)		

*IFNL4* Interferon lambda 4, *TLR2* Toll-like receptors, *χ*^2^ Chi square test, *HW* Hardy–Weinberg equilibrium, ® Reference group, *p* *p* value, *Statistically significant at *p* < 0.05

**Table 5:** Comparison between the four studied groups according to different SNPs

	Patients			Control (*n* = 74)	Sig. bet. groups					
	Non-severe (*n* = 78)	Severe (*n* = 39)	Critical (*n* = 33)		p_1_	p_2_	p_3_	p_4_	p_5_	p_6_
*FURIN*										
G/G®	30 (38.5%)	6 (15.4%)	4 (12.1%)	37 (50%)	0.011*	0.023*	0.272	0.392	< 0.001*	0.001*
G/C	30 (38.5%)	15 (38.5%)	18 (54.5%)	26 (35.1%)						
C/C	18 (23.1%)	18 (46.2%)	11 (33.3%)	11 (14.9%)						
^HW^p	0.061	0.348	0.414	0.088						
*Allele*										
G®	90 (57.7%)	27 (34.6%)	26 (39.4%)	100 (67.6%)	0.001*	0.013*	0.075	0.554	< 0.001*	< 0.001*
C	66 (42.3%)	51 (65.4%)	40 (60.6%)	48 (32.4%)						
*Dominant genetic model*										
G/G	30 (38.5%)	6 (15.4%)	4 (12.1%)	37 (50.0%)	0.011*	0.006*	0.152	0.690	< 0.001*	< 0.001*
G/C + C/C	48 (61.5%)	33 (84.6%)	29 (87.9%)	37 (50.0%)						
*Recessive genetic model*										
G/G + G/C	60 (76.9%)	21 (53.8%)	22 (66.7%)	63 (85.1%)	0.011*	0.261	0.198	0.269	< 0.001*	0.029*
C/C	18 (23.1%)	18 (23.1%)	11(33.3%)	11 (14.9%)						
*IFNL4*										
C/C®	33 (42.3%)	13 (33.3%)	8 (24.2%)	39 (52.7%)	0.038*	0.003*	0.419	0.641	0.020*	0.001*
C/T	36 (46.2%)	14 (35.9%)	12 (36.4%)	27 (36.5%)						
T/T	9 (11.5%)	12 (30.8%)	13 (39.4%)	8 (10.8%)						
^HW^p	0.863	0.079	0.142	0.323						
*Allele*										
C®	102 (65.4%)	40 (51.3%)	28 (42.4%)	105 (70.9%)	0.037*	0.002*	0.298	0.289	0.003*	< 0.001*
T	54 (34.6%)	38 (48.7%)	38 (57.6%)	43 (29.1%)						
*Dominant genetic model*										
C/C	33 (42.3%)	13(33.3%)	8 (24.2%)	39 (52.7%)	0.349	0.071	0.200	0.398	0.49*	0.006*
C/T + T/T	45 (57.7%)	26 (66.7%)	25 (75.8%)	35 (47.3%)						
*Recessive genetic model*										
C/C + C/T	69 (88.5%)	27 (69.2%)	20 (60.6%)	66 (89.2%)	0.011*	0.001*	0.887	0.444	0.008*	0.001*
T/T	9 (11.5%)	12 (30.8%)	13 (39.4%)	8 (10.8%)						
*TLR2*										
T/T®	24 (30.8%)	6 (15.4%)	5 (15.2%)	36 (48.6%)	0.012*	0.004*	0.077	0.888	< 0.001*	< 0.001*^*^
T/C	38 (48.7%)	15 (38.5%)	11 (33.3%)	26 (35.1%)						
C/C	16 (20.5%)	18 (46.2%)	17 (51.5%)	12 (16.2%)						
^HW^p	0.893	0.348	0.183	0.065						
*Allele*										
T®	86 (55.1%)	27 (34.6%)	21 (31.8%)	98 (66.2%)	0.003*	0.001*	0.048*	0.723	< 0.001*	< 0.001*
C	70 (44.9%)	51 (65.4%)	45 (68.2%)	50 (33.8%)						
*Dominant genetic model*										
T/T	24 (30.8%)	6 (15.4%)	5 (15.2%)	36 (48.6%)	0.072	0.087	0.024*	0.978	0.001*	0.001*
T/C + C/C	54 (69.2%)	33 (84.6%)	28 (84.8%)	38 (51.4%)						
*Recessive genetic model*										
T/T + T/C	62 (79.5%)	21 (53.8%)	16 (48.5%)	62 (83.8%)	0.004*	0.001*	0.495	0.650	0.001*	< 0.001*
C/C	16 (20.5%)	18 (46.2%)	17 (51.5%)	12 (16.2%)						

*IFNL4* Interferon lambda 4, *TLR2* Toll-like receptors, *χ*^2^ Chi square test, *HW* Hardy–Weinberg equilibrium, ® Reference group, *p* *p* value, *p*_1_ *p* value for comparing between non-severe and severe, p_2_ *p* value for comparing between non-severe and critical, p_3_ *p* value for comparing between non-severe and controls, p_4_ *p* value for comparing between severe and critical, p_5_ *p* value for comparing between severe and controls, p_6_ *p* value for comparing between critical and controls, *Statistically significant at *p* < 0.05

Table 6 displays the frequency distribution of genotypes, genotype groups, and alleles of the analyzed *FURIN*, *IFNL4*, and *TLR2* polymorphisms among COVID-19 patients and controls. Importantly, for *FURIN* rs6226, we noticed that carriers of rs6226 G/G variant homozygous genotypes were less likely to develop COVID-19 disease. In addition, (G/C + C/C) genotypes were significantly more frequent in COVID‐19 patients compared to the control group (OR: 2.750, CI 1.537–4.921). Also, the (C/T + T/T) genotype for *IFNL4* rs12979860 (p = 0.018, OR = 1.981, CI 1.126–3.486) and (T/C + C/C) genotypes for *TLR2* rs3804099 (*p* < 0.001, OR = 3.113, CI 1.721–5.629) showed similar distribution. Multivariable logistic regression analysis showed that age and dominant genetic models for *FURIN* and *TLR2* were independently associated with COVID-19 susceptibility (ORs: 1.125, 3.293, and 2.839, respectively), as demonstrated in Table 7.

**Table 6:** Frequencies of different SNPs genotype & allele among COVID-19 cases compared to controls

	Total patient (*n* = 150)	Control® (*n* = 74)	OR	
			*p*	(LL–UL 95%C.I)
*FURIN*				
G/G®	40 (26.7%)	37 (50%)		1.000
G/C	63 (42%)	26 (35.1%)	0.013*	2.241 (1.183–4.247)
C/C	47 (31.3%)	11 (14.9%)	0.001*	3.952 (1.786–8.745)
*Allele*				
G®	143 (47.7%)	100 (67.6%)		1.000
C	157 (52.3%)	48 (32.4%)	< 0.001*	2.287 (1.515–3.454)
*Dominant genetic model*				
G/G	40 (26.7%)	37 (50.0%)		1.000
G/C + C/C	110 (73.3%)	37 (50.0%)	0.001*	2.750 (1.537–4.921)
*Recessive genetic model*				
G/G + G/C	103 (68.7%)	63 (85.1%)		1.000
C/C	47 (31.3%)	11 (14.9%)	0.010*	2.613 (1.263–5.409)
*Log additive genetic model*				
G/G	40 (26.7%)	37 (50%)		
G/C	63 (42%)	26 (35.1%)	< 0.001*	2.028(1.373–2.995)
C/C	47 (31.3%)	11 (14.9%)		
*IFNL4*				
C/C®	54 (36%)	39 (52.7%)		1.000
C/T	62 (41.3%)	27 (36.5%)	0.105	1.658 (0.900–3.057)
T/T	34 (22.7%)	8 (10.8%)	0.012*	3.069 (1.282–7.351)
*Allele*				
C®	170 (56.7%)	105 (70.9%)		1.000
T	130 (43.3%)	43 (29.1%)	0.004*	1.867 (1.224–2.848)
*Dominant genetic model*				
C/C	54 (36.0%)	39 (52.7%)		1.000
C/T + T/T	96 (64.0%)	35 (47.3%)	0.018*	1.981 (1.126–3.486)
*Recessive genetic model*				
C/C + C/T	116 (77.3%)	66 (89.2%)		1.000
T/T	34 (22.7%)	8 (10.8%)	0.036*	2.418 (1.057–5.531)
*Log additive genetic model*				
C/C	54 (36%)	39 (52.7%)		
C/T	62 (41.3%)	27 (36.5%)	0.008*	1.727(1.157–2.577)
T/T	34 (22.7%)	8 (10.8%)		
*TLR2*				
T/T®	35 (23.3%)	36 (48.6%)		1.000
T/C	64 (42.7%)	26 (35.1%)	0.005*	2.532 (1.320–4.856)
C/C	51 (34%)	12 (16.2%)	< 0.001*	4.371 (1.999–9.558)
*Allele*				
T®	134 (44.7%)	98 (66.2%)		1.000
C	166 (55.3%)	50 (33.8%)	< 0.001*	2.428 (1.612–3.657)
*Dominant genetic model*				
T/T	35 (23.3%)	36 (48.6%)		1.000
T/C + C/C	115 (76.7%)	38 (51.4%)	< 0.001*	3.113 (1.721–5.629)
*Recessive genetic model*				
T/T + T/C	99 (66.0%)	62 (83.8%)		
C/C	51 (34.0%)	12 (16.2%)	0.006*	2.662 (1.316–5.384)
*Log additive genetic model*				
T/T	35 (23.3%)	36 (48.6%)		
T/C	64 (42.7%)	26 (35.1%)	< 0.001*	2.145(1.451–3.170)
C/C	51 (34%)	12 (16.2%)		

*IFNL4* Interferon lambda 4, *TLR2* Toll-like receptors, *OR* Odd’s ratio, *AOR* Adjusted Odd`s ratio, ® Reference group, *CI* Confidence interval, *LL* Lower limit, *UL* Upper Limit, *p* *p* value, *Statistically significant at *p* < 0.05, ^#^Adjusted with age and sex

**Table 7:** Multivariable logistic regression regarding susceptibility to COVID‐19 infection

	B	SE	Sig	OR	95% CI	
					LL	UL
(Constant)	– 6.251	0.998	< 0.001*	0.002		
Age	0.118	0.020	< 0.001*	1.125	1.081	1.171
Sex (Male)	– 0.231	0.366	0.529	0.794	0.387	1.628
*FURIN* (Dominant genetic model)	1.192	0.389	0.002*	3.293	1.537	7.056
*IFNL4* (Dominant genetic model)	0.427	0.364	0.241	1.533	0.751	3.127
*TLR2* (Dominant genetic model)	1.044	0.394	0.008*	2.839	1.312	6.145

*IFNL4* Interferon lambda 4, *TLR2* Toll-like receptors, *B* Unstandardized Coefficients, *SE* standard error, *OR* Odds ratio, *C.I* Confidence interval, *LL* Lower limit, *UL* Upper limit, *Statistically significant at *p* < 0.05

Tables 8 and 9 illustrate the role of the investigated gene polymorphisms in the progression of COVID-19 disease and the predictors of progression to severe and critical illness. For *FURIN* rs6226, we found that (G/C + C/C) represented 62 (86.1%) of severe and critical cases compared to 48 (61.5%) of non-severe ones with a significant difference (*p* = 0.001, OR = 3.875, CI 1.726–8.700). Furthermore, T/T homozygosity of IFNL4 rs12979860 was detected in 25 (34.7%) in severe and critical cases compared to 9 (11.5%) in those with non-severe illness (*p* = 0.001, OR = 4.078, CI 1.748–9.515). Regarding TLR2 rs3804099, C/C homozygosity was significantly more frequent in severe and critically ill patients (*p* = 0.001, OR = 3.666, CI 1.788–7.516). On multivariate analysis, age, presence of DM or hypertension, and recessive genetic models of *FURIN* and *IFNL4* were the independent risk factors for severe and critical illness (ORs (CI) 1.054 (1.02–1.088), 4.162 (1.596–10.85), 3.724 (1.337–10.374), 3.041 (1.139–8.123), and 3.420 (1.152–10.153), respectively.

**Table 8:** Frequencies of different SNPs genotype & allele according to severity of COVID-19 infection

	Severe/ critical (*n* = 72)	Non-severe® (*n* = 78)	OR	
			*p*	(LL–UL 95%C.I)
*FURIN*				
G/G®	10 (13.9%)	30 (38.5%)		1.000
G/C	33 (45.8%)	30 (38.5%)	0.007*	3.300 (1.383–7.876)
C/C	29 (40.3%)	18 (23.1%)	0.001*	4.833 (1.914–12.205)
*Allele*				
G®	53 (36.8%)	90 (57.7%)		1.000
C	91 (63.2%)	66 (42.3%)	< 0.001*	2.341 (1.472–3.725)
*Dominant genetic model*				
G/G	10 (13.9%)	30 (38.5%)		1.000
G/C + C/C	62 (86.1%)	48 (61.5%)	0.001*	3.875 (1.726–8.700)
*Recessive genetic model*				
G/G + G/C	43 (59.7%)	60 (76.9%)		1.000
C/C	29 (40.3%)	18 (23.1%)	0.025*	2.248 (1.109–4.557)
*Log additive genetic model*				
G/G	10 (13.9%)	30 (38.5%)		
G/C	33 (45.8%)	30 (38.5%)	0.001*	2.134(1.357–3.355)
C/C	29 (40.3%)	18 (23.1%)		
*IFNL4*				
C/C®	21 (29.2%)	33 (42.3%)		1.000
C/T	26 (36.1%)	36 (46.2%)	0.739	1.135 (0.539–2.389)
T/T	25 (34.7%)	9 (11.5%)	0.002*	4.365 (1.709–11.152)
*Allele*				
C®	68 (47.2%)	102 (65.4%)		1.000
T	76 (52.8%)	54 (34.6%)	0.002*	2.111 (1.327–3.360)
*Dominant genetic model*				
C/C	21 (29.2%)	33 (42.3%)		1.000
C/T + T/T	51 (70.8%)	45 (57.7%)	0.095	1.781 (0.904–3.509)
*Recessive genetic model*				
C/C + C/T	47 (65.3%)	69 (88.5%)		1.000
T/T	25 (34.7%)	9 (11.5%)	0.001*	4.078 (1.748–9.515)
*Log additive genetic model*				
C/C	21 (29.2%)	33 (42.3%)		
C/T	26 (36.1%)	36 (46.2%)	0.004*	1.936(1.237–3.028)
T/T	25 (34.7%)	9 (11.5%)		
*TLR2*				
T/T®	11 (15.3%)	24 (30.8%)		1.000
T/C	26 (36.1%)	38 (48.7%)	0.367	1.493 (0.625–3.566)
C/C	35 (48.6%)	16 (20.5%)	0.001*	4.773 (1.889–12.059)
*Allele*				
T®	48 (33.3%)	86 (55.1%)		1.000
C	96 (66.7%)	70 (44.9%)	< 0.001*	2.457 (1.538–3.926)
*Dominant genetic model*				
T/T	11 (15.3%)	24 (30.8%)		1.000
T/C + C/C	61 (84.7%)	54 (69.2%)	0.028*	2.465 (1.105–5.497)
*Recessive genetic model*				
T/T + T/C	37 (51.4%)	62 (79.5%)		1.000
C/C	35 (48.6%)	16 (20.5%)	< 0.001*	3.666 (1.788–7.516)
*Log additive genetic model*				
T/T	11 (15.3%)	24 (30.8%)		
T/C	26 (36.1%)	38 (48.7%)	0.001*	2.272(1.427–3.616)
C/C	35 (48.6%)	16 (20.5%)		

*IFNL4* Interferon lambda 4, *TLR2* Toll-like receptors, *OR* Odd’s ratio, *AOR* Adjusted Odd`s ratio, ® Reference group, *CI* Confidence interval, *LL* Lower limit, *UL* Upper Limit, *p* *p* value, *Statistically significant at *p *< 0.05, # Adjusted with Age, Sex, DM and HTN

**Table 9:** Multivariate regression analysis regarding severity of COVID 19 infection

	B	SE	Sig	OR	95% CI	
					LL	UL
(Constant)	– 3.924	0.846	0.000	0.020		
Age	0.053	0.016	0.001*	1.054	1.021	1.088
Sex (Male)	– 0.736	0.498	0.140	0.479	0.181	1.272
DM	1.426	0.489	0.004*	4.162	1.596	10.854
Hypertension	1.315	0.523	0.012*	3.724	1.337	10.374
*FURIN* (Dominant genetic model)	1.112	0.501	0.027*	3.041	1.139	8.123
*IFNL4* (Recessive genetic model)	1.230	0.555	0.027*	3.420	1.152	10.153
*TLR2* (Recessive genetic model)	0.823	0.481	0.087	2.277	0.887	5.847

*IFNL4* Interferon lambda 4, *TLR2* Toll-like receptors, *B* Unstandardized Coefficients, *SE* standard error, *OR* Odds ratio, *CI* Confidence interval, *LL* Lower limit, *UL* Upper limit, *Statistically significant at *p* < 0.05

Thirty patients (20%) out of 150 COVID-19-included patients passed away. Supplementary Tables 1 and 2 show the clinical, laboratory, and demographic characteristics of alive and dead patients. We found that 90.0% of dead patients, compared to 69.2% of survived patients, had *FURIN* (G/C + C/C) genotypes (*p* = 0.021). For *IFNL4,* T/T homozygous was found in 46.7% and 16.7% of dead and survived patients, respectively (*p* < 0.001). In addition, the C/C genotype of *TLR2* was significantly more frequent in dead patients (63.3%) (Table 10). Multivariate logistic regression analysis revealed that age, history of DM or hypertension, blood urea, serum creatinine, *FURIN* (G/C + C/C) genotypes, and *IFNL4* T/T homozygous were independent risk factors associated with increased mortality (Table 11).

**Table 10:** Relation between outcome and different SNPs genotype & allele

	Outcome		χ^2^	*p*
	Alive (*n* = 120)	Died (*n* = 30)		
*FURIN*				
G/G	37 (30.8%)	3 (10%)	8.230*	0.016*
G/C	44 (36.7%)	19 (63.3%)		
C/C	39 (32.5%)	8 (26.7%)		
*Allele*				
G	118 (49.2%)	25 (41.7%)	1.082	0.298
C	122 (50.8%)	35 (58.3%)		
*Dominant genetic model*				
G/G	37 (30.8%)	3 (10.0%)	5.327*	0.021*
G/C + C/C	83 (69.2%)	27 (90.0%)		
*Recessive genetic model*				
G/G + G/C	81 (67.5%)	22 (73.3%)	0.380	0.538
C/C	39 (32.5%)	8 (26.7%)		
*IFNL4*				
C/C	46 (38.3%)	8 (26.7%)	12.388*	0.002*
C/T	54 (45%)	8 (26.7%)		
T/T	20 (16.7%)	14 (46.7%)		
*Allele*				
C	146 (60.8%)	24 (40%)	8.484*	0.004*
T	94 (39.2%)	36 (60%)		
*Dominant genetic model*				
C/C	46 (38.3%)	8 (26.7%)	1.418	0.234
C/T + T/T	74 (61.7%)	220(73.3%)		
*Recessive genetic model*				
C/C + C/T	100 (83.3%)	16 (53.3%)	12.323*	< 0.001*
T/T	20 (16.7%)	14 (46.7%)		
*TLR2*				
T/T	33 (27.5%)	2 (6.7%)	15.365*	< 0.001*
T/C	55 (45.8%)	9 (30%)		
C/C	32 (26.7%)	19 (63.3%)		
*Allele*				
T	121 (50.4%)	13 (21.7%)	16.053*	< 0.001*
C	119 (49.6%)	47 (78.3%)		
*Dominant genetic model*				
T/T	33 (27.5%)	2 (6.7%)	5.823*	0.016*
T/C + C/C	87 (72.5%)	28 (93.3%)		
*Recessive genetic model*				
T/T + T/C	88 (73.3%)	11 (36.7%)	14.379*	< 0.001*
C/C	32 (26.7%)	19 (63.3%)		

*IFNL4* Interferon lambda 4, *TLR2* Toll-like receptors, *χ2* Chi square test, *p* *p* value, *Statistically significant at *p* < 0.05

**Table 11:** Univariate and multivariate logistic regression analysis predicting mortality

	Univariate		^#^Multivariate	
	*p*	OR (LL–UL 95%C.I)	*p*	OR (LL–UL 95%C.I)
Age	< 0.001*	1.078 (1.044–1.114)	0.008^*^	1.102 (1.025–1.185)
Sex	0.367	0.684 (0.300–1.561)		
DM	< 0.001*	13.500 (3.878–47.0)	0.028*	0.112 (0.016–0.789)
Hypertension	< 0.001*	6.667 (2.532–17.550)	0.047*	0.124 (0.016–0.973)
CO-RADS	0.995	–		
Urea	0.001^*^	1.034 (1.013–1.055)	0.002*	1.100 (1.036–1.168)
Creatinine	0.025^*^	5.026 (1.227–20.584)	0.009*	0.004 (0.0–0.254)
ALT	0.008^*^	1.015 (1.004–1.027)	0.720	1.004 (0.981–1.028)
PT	0.203	1.284 (0.874–1.888)		
INR	0.147	0.640 (0.349–1.171)		
D-dimer	0.007^*^	1.875 (1.192–2.949)	0.077	0.416 (0.157–1.101)
Prolcalcitonin	< 0.001*	5.486 (2.391–12.586)	0.606	1.616 (0.261–10.023)
IL-6	0.052	1.005 (1.0–1.011)		
*FURIN* (Dominant genetic model)	0.030^*^	4.012 (1.145–14.062)	0.027*	12.103 (1.329–110.243)
*IFNL4* (Recessive genetic model)	0.001^*^	4.375 (1.846–10.371)	0.256	2.656 (0.492–14.342)
*TLR2* (Recessive genetic model)	< 0.001*	4.750 (2.039–11.065)	0.001*	19.854 (3.310–119.087)

*IFNL4* Interferon lambda 4, *TLR2* Toll-like receptors, *OR* Odd`s ratio, *CI* Confidence interval, *LL* Lower limit, *UL* Upper limit, ^#^ All variables with *p* < 0.05 was included in the multivariable, *Statistically significant at *p* ≤ 0.05

**Fig. 1:**
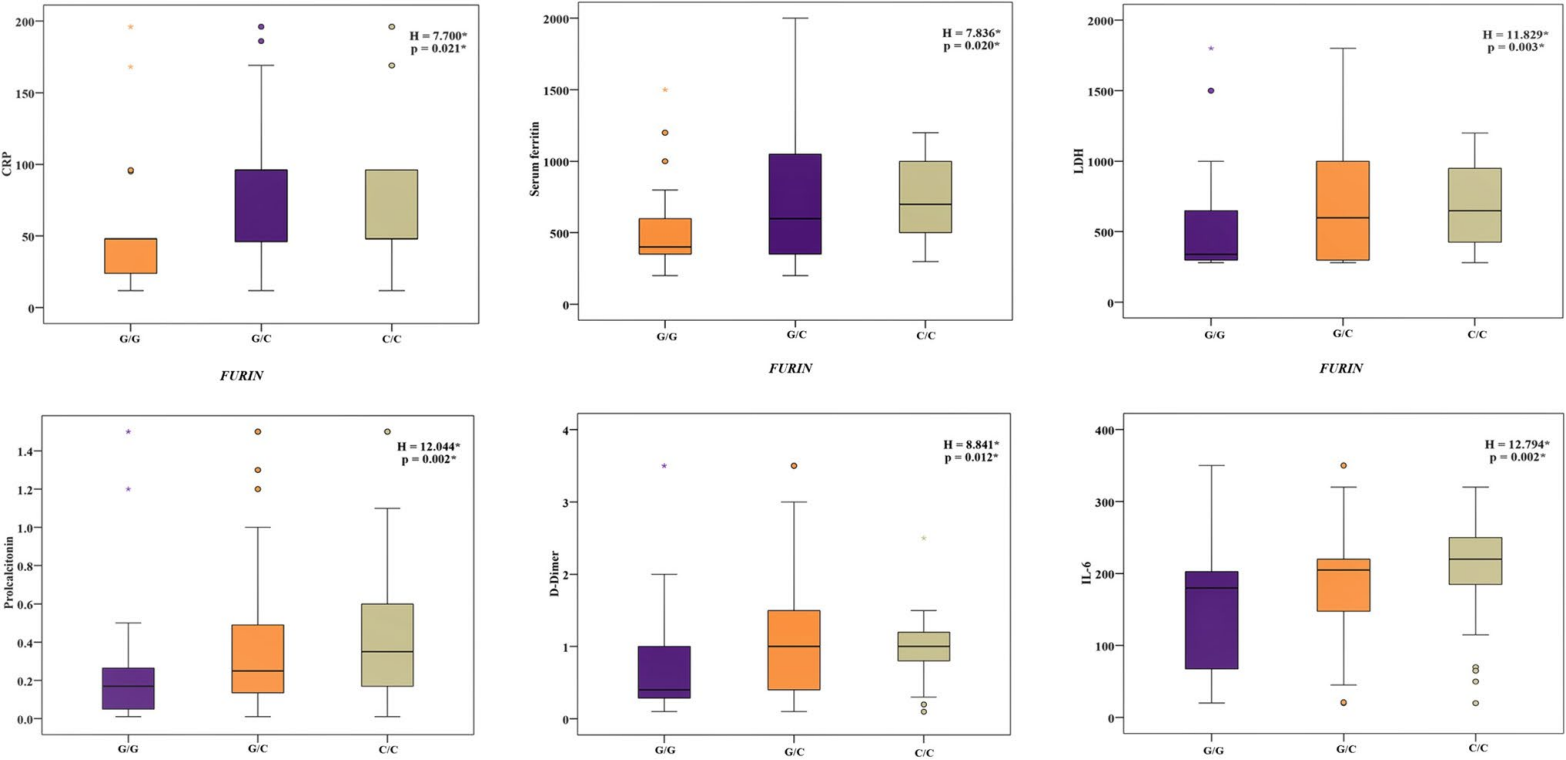
Relation between *FURIN* genotypes and different inflammatory parameters in patients group

**Fig. 2:**
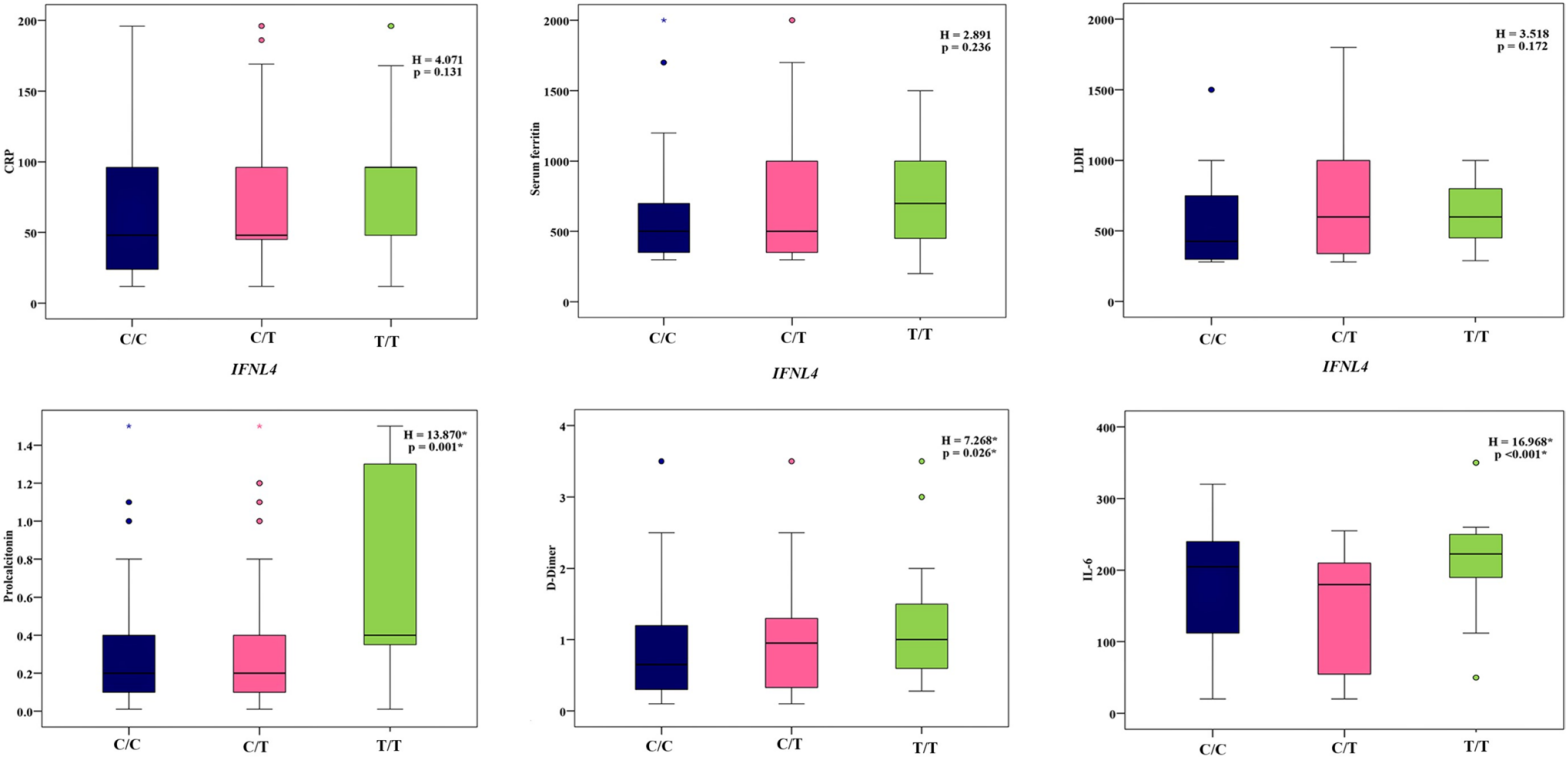
Relation between *IFNL4* genotypes and different inflammatory parameters in patients group

**Fig. 3:**
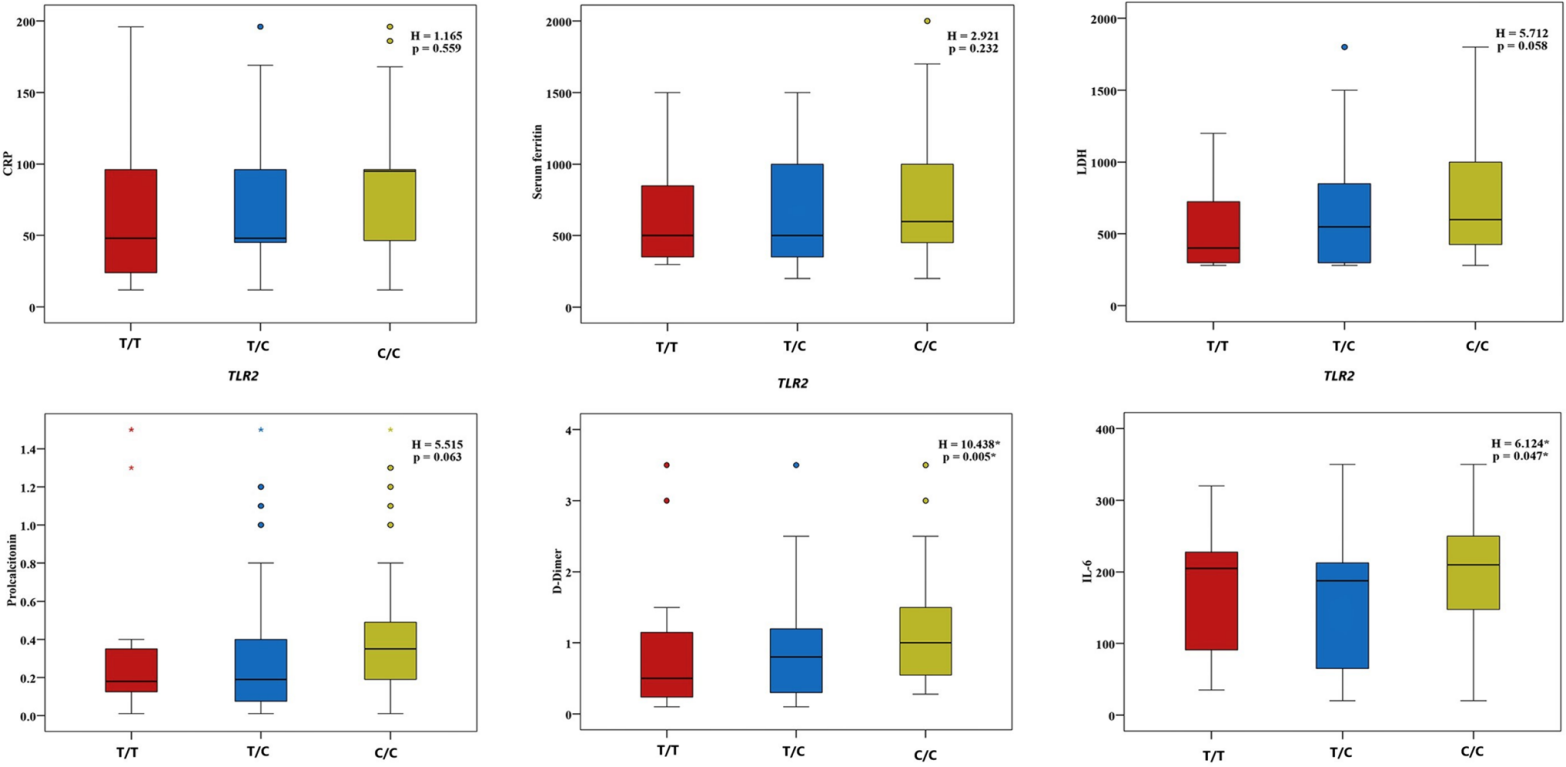
Relation between TLR2 genotypes and different inflammatory parameters in patients group

Regarding the relation between the studied genes and laboratory findings, *FURIN* C/C and G/C genotypes were significantly associated with higher values of segmented neutrophils (*P* = 0.045), CRP (*P* = 0.021), LDH (*P* = 0.003), serum ferritin (*P* = 0.020), procalcitonin (*P* = 0.002), D-dimer (*P* = 0.012), and IL6 (*P* = 0.002) (Fig. 1; Supplementary Table 3). *IFNL4* genotypes showed no variation except with ALT (*P* = 0.004), PT (*P* = 0.031), procalcitonin (*P* = 0.001), D-dimer (*P* = 0.026), and IL-6 levels, where the T/T genotype was linked to their highest values (Fig. 2; Supplementary Table 4). The C/C and T/C genotypes of *TLR2* were correlated with the highest D-dimer values (*P* = 0.005) (Fig. 3; Supplementary Table 5).

## Discussion

A better understanding of SARS-CoV-2 direct and indirect damage in the host is required. Numerous studies on the effect of host genetics on COVID-19 infection and clinical outcomes have focused on virus receptor genetic variants and infection susceptibility [17]. However, there is little information on some other genes associated with disease pathology, particularly in Egypt and the Middle East. Genome-wide association studies (GWAS) discovered numerous associations between both coding and non-coding variants and disease. Protein-coding variants, with loss or gain of function mutations, can disrupt normal protein function and exert detrimental effects on a phenotype [18]. The COVID-19 associations lie primarily in the non-coding region of the genome, and their functions are still not clear [19]. Non-coding variants have no immediate effect on protein function and are significantly enriched in functional non-coding regions such as enhancer elements, DNase hypersensitivity regions, and chromatin marks. These variants can lead to clinical conditions and affect the course of the disease through indirect regulation of gene expression, controlling DNA replication timing, gene interruption, gene fusion, and other effects on gene function [20].

Unfortunately, Egypt’s published data about the role of host genetics in COVID-19 is restricted. Thereby, the present study aimed to investigate the association of three substantial gene polymorphisms (*FURIN*, *IFN4*, and *TLR2*) with COVID-19 susceptibility and severity in Egyptian patients. We found that older people are more affected and more likely to develop more severe illnesses, in line with previous findings [21, 22]. Also, diabetes and hypertension were common in COVID-19 patients, especially in severely and critically ill patients, as previously reported [23, 24]. A significantly increased ALT level as well as higher inflammatory indices (CRP, LDH, ferritin, and procalcitonin) and IL-6 levels were detected in COVID-19 patients compared to controls, with the highest values in severe and critically ill patients. Liver enzyme elevation, notably increased ALT activity, has been observed mainly in severe COVID-19 patients and is associated with a more negative outcome [25]. SARS-CoV-2 itself can cause direct liver damage by penetrating the liver tissue through its receptor or indirect damage through multisystem inflammation [26]. It has been well known that the pulmonary inflammation related to COVID-19 infection is linked to high levels of certain proinflammatory cytokines, including interleukin-6 (IL-6) [27].

In the current study, *FURIN* rs6226 genotypes G/C and C/C, as well as the C allele, were all more frequent in patients than controls and were also the most prevalent in severely and critically ill patients. We found that *FURIN*-dominant genetic models were independent predictors of COVID-19 susceptibility, severity, and mortality. A previous study found several variants in the *FURIN* gene, including rs6226. However, none of them was associated with COVID‐19 in Madrid, Spain [28]. Another study detected deleterious variants in *FURIN*, also including rs6226, suggesting a decrease in FURIN protease function that potentially can reduce the risk of SARS-CoV-2, which may explain COVID-19 clinical disparity in Middle Eastern populations (Kuwait, Qatar, and Iran) [7]. *FURIN* variants could clarify the heterogeneous response to SARS-CoV-2 infection, as variants that significantly raise *FURIN* expression may be linked to poor outcomes. Moreover, FURIN has been associated with an increased risk of cardiovascular traits such as diabetes, hypercholesterolemia, and hypertension, all of which are risk factors for COVID-19 severity and mortality [29].

We noticed that *IFNL4* rs12979860 genotypes and its alleles differed significantly between patients with severe or critical illness and those with non-severe presentation (Fig. 3). The C/C protective genotype was more frequent in non-severe cases and controls, while the T/T risky genotype was more frequent in severe and critical cases. However, no variation was detected between the non-severe and control groups, as well as between the severe and critical groups. It has been reported that the co-expression of *IFNL4* C/C was a powerful predictor of resistance to COVID-19 infection [30]. We also demonstrated that the presence of the T/T genotype and the T allele for *IFNL4* were significantly associated with increased mortality in COVID-19 patients. In line with our finding, Agwa et al. declared that the T allele was linked to the existence of comorbidities and an elevated mortality rate in comparison with other genotypes [31]. Another study also reported that the T allele of rs12979860 was significantly overexpressed in COVID-19 patients [10]. Amodio et al. indicated a lower ability for viral clearance in individuals who had the rs1297860 T/T genotype [30]. Contrary to our findings, a previous study reported the protective role of the IFNL4 minor allele, as carriers of the *IFNL4* T allele were less likely to progress from mild to moderate COVID‐19 [32]. Also, another study found that the *IFNL4* T/T genotype and T allele were significantly associated with a lower likelihood of fatal outcome [33].

A recent study reported that *TLR2* rs5743708 variants have been related to a higher risk and severity of COVID-19 infection [34]. However, data about the role of the *TLR2* rs3804099 SNP in COVID-19 is lacking. We reported that T/C and C/C variants and the C allele of *TLR2* rs3804099 were more prevalent in patients than controls. Additionally, the *TLR2* C allele was significantly more frequent in severe and critical stages and was associated with higher mortality rates. Conti et al. proposed that the activation of *TLR2* during COVID-19 infection could result in the production of proinflammatory cytokines like IL-1, and its interaction with the virus particles causes immunopathological consequences that lead to death in COVID-19 patients [35]. It was reported that cytokine production (IL-10, IL-8, and TNF-α) was significantly increased in patients with the TLR2 rs3804099 C allele compared with those with the T allele in response to bacterial lipoprotein stimulation [36]. Regarding its role in viral diseases, it was found that the rs3804099 CT and TT genotypes had a protective effect on the progression of hepatitis B and C and were associated with inhibiting IL-6 and TNF-α levels. In contrast, the haplotype CC significantly increased disease progression. However, the role of this genetic variant in COVID-19 susceptibility and severity is still unclear [37].

We studied the relationship between *FURIN*, *IFNL4*, and *TLR2* genotypes and laboratory parameters in COVID-19 patients, particularly ALT as a marker of liver injury. Only *IFNL4* genotypes showed a significant correlation with ALT, with T/T and C/T genotypes linked to the highest values. *IFNL4* T/T and C/T genotypes were also significantly correlated with PT, procalcitonin, and D-dimer. *FURIN* C/C and G/C genotypes were significantly associated with higher neutrophils, CRP, LDH, ferritin, procalcitonin, D-dimer, and IL-6. *TLR2* C/C and T/C genotypes were correlated with higher D-dimer only. Although *TLR2* rs3804099 is commonly associated with the degree of liver injury in viral liver disease**s** [12], no significant correlation was found between *TLR2* rs3804099 and ALT levels.

Our study is limited by the small number of patients and the fact that it is a single-center study. Also, many functional SNPs that are in high linkage disequilibrium with *FURIN*, *IFNL4,* and *TLR2* loci are not tested. Furthermore, data on some risk factors, such as environmental and socioeconomic variables, as well as treatments used, is missing. Larger multicenter studies are needed to adequately evaluate the significance of the studied SNPs in COVID-19 patients.

In conclusion, *FURIN*, *IFNL4*, and *TLR2* gene variants are associated with the risk of COVID-19 occurrence and linked to increased severity and worse outcomes in Egyptian patients. Confirmation of these results is still needed to help in further studies.

## Supplementary Information

Below is the link to the electronic supplementary material.

Supplementary file1 (DOCX 49 KB)

## Data Availability

All data generated or analyzed during the current study are included in this published article.

## Abbreviations

IL-28: Interleukin-28
TLR2: Toll-like receptor 2
COVID-19: Coronavirus disease 2019
SARS-CoV-2: Coronavirus 2 severe acute respiratory syndrome
IFNL3: Interferon lambda type 3
SNPs: Single nucleotide polymorphisms
PCR: Polymerase chain reaction
DM: Diabetes mellitus
HTN: Hypertension
WHO: World Health Organization
CBC: Complete blood count
Hb: Hemoglobin concentration
WBCs: White blood cells
CRP: C reactive protein
ALT: Alanine transaminase
LDH: Lactate dehydrogenase
PT: Prothrombin time
INR: International normalized ratio
IL-6: Interleukin-6
RNA: Ribonucleic acid
