# Supplementary material for: FURIN, IFNL4, and TLR2 gene polymorphisms in relation to COVID-19 severity: a case–control study in Egyptian patients
Source: Infection. 2024 May 4;52(6):2213–29. doi: 10.1007/s15010-024-02266-1 (PMC11621141; doi:10.1007/s15010-024-02266-1)
Supplement: Supplementary file 1 — Supplementary file1 (DOCX 49 KB) [file 15010_2024_2266_MOESM1_ESM.docx]

**Supplementary table 1: Relation between outcome, demographic and laboratory data**

|  | **Outcome** | | **Test of Sig.** | **p** |
| --- | --- | --- | --- | --- |
|  | **Alive (n = 120)** | **Died (n = 30)** |  |  |
| **Sex** |  |  |  |  |
| Male | 65 (54.2%) | 19 (63.3%) | **χ^2^**= 0.818 | 0.366 |
| Female | 55 (45.8%) | 11 (36.7%) |  |  |
| **Age (years)** |  |  |  |  |
| Mean ± SD. | 54.0 ± 14.8 | 70.6 ± 15.6 | U= 789.00^*^ | >0.001^*^ |
| Median (Min. – Max.) | 53 (25 – 80) | 75 (42 – 88) |  |  |
| **Hb (g/dL)** |  |  |  |  |
| Mean ± SD. | 11.1 ± 1.53 | 10.8 ± 1.86 | U= 1665.00 | 0.523 |
| Median (Min. – Max.) | 11 (8.50 – 14) | 11 (7.50 – 14) |  |  |
| **WBCs (×1000/ µL)** |  |  |  |  |
| Mean ± SD. | 8.36 ± 4.17 | 13.7 ± 9.16 | U= 1240.500^*^ | 0.008^*^ |
| Median (Min. – Max.) | 8.75 (3.80 – 25) | 10 (4 – 32) |  |  |
| **Lymphocytes (%)** |  |  |  |  |
| Mean ± SD. | 14.3 ± 3.82 | 12.5 ± 5.06 | U= 1434.00 | 0.084 |
| Median (Min. – Max.) | 13.5 (5 – 23) | 12 (5 – 20) |  |  |
| **Segmented (%)** |  |  |  |  |
| Mean ± SD. | 78.2 ± 8.13 | 83.7 ± 5.64 | U= 1044.00^*^ | >0.001^*^ |
| Median (Min. – Max.) | 79 (38 – 90) | 81.5 (75 – 90) |  |  |
| **Platelet count (×1000/ µL)** |  |  |  |  |
| Mean ± SD. | 255.4 ± 74.8 | 247.2 ± 107.7 | U= 1482.00 | 0.135 |
| Median (Min. – Max.) | 235 (129 – 430) | 190.5 (140 – 450) |  |  |
| **CRP(mg/L)** |  |  |  |  |
| Mean ± SD. | 65.4 ± 50.3 | 112.1 ± 52.7 | U= 805.500^*^ | >0.001^*^ |
| Median (Min. – Max.) | 48 (12 – 196) | 96 (48 – 196) |  |  |
| **Serum Creatinine (mg/dL)** | |  |  |  |
| Mean ± SD. | 1.06 ± 0.23 | 1.18 ± 0.31 | U= 1345.500^*^ | 0.027^*^ |
| Median (Min. – Max.) | 1 (0.80 – 2.10) | 1.10 (0.80 – 1.90) |  |  |
| **Blood urea (mg/dL)** |  |  |  |  |
| Mean ± SD. | 41.6 ± 13.8 | 54.4 ± 26.7 | U= 1356.00^*^ | 0.035^*^ |
| Median (Min. – Max.) | 40 (25 – 100) | 45 (25 – 102) |  |  |
| **ALT (IU/L)** |  |  |  |  |
| Mean ± SD. | 38.1 ± 23.5 | 63.3 ± 65.2 | U= 1117.500^*^ | 0.001^*^ |
| Median (Min. – Max.) | 30 (18 – 120) | 44 (20 – 250) |  |  |
| **Serum ferritin (ng/mL)** |  |  |  |  |
| Mean ± SD. | 616.1 ± 353.8 | 941.7 ± 506.4 | U= 993.00^*^ | >0.001^*^ |
| Median (Min. – Max.) | 500 (200 – 1700) | 750 (200 – 2000) |  |  |
| **LDH (IU/L)** |  |  |  |  |
| Mean ± SD. | 580.5 ± 317.4 | 923 ± 473.5 | U= 984.00^*^ | >0.001^*^ |
| Median (Min. – Max.) | 450 (280 – 1500) | 750 (290 – 1800) |  |  |
| **D-Dimer (mg/L)** |  |  |  |  |
| Mean ± SD. | 0.91 ± 0.79 | 1.38 ± 0.81 | U= 1069.500^*^ | 0.001^*^ |
| Median (Min. – Max.) | 0.80 (0.10 – 3.50) | 1 (0.50 – 3.50) |  |  |
| **Prolcalcitonin (ng/mL)** |  |  |  |  |
| Mean ± SD. | 0.33 ± 0.39 | 0.73 ± 0.53 | U= 898.500^*^ | >0.001^*^ |
| Median (Min. – Max.) | 0.19 (0.01 – 1.50) | 0.49 (0.01 – 1.50) |  |  |
| **PT(seconds)** |  |  |  |  |
| Mean ± SD. | 13.1 ± 1.01 | 13.4 ± 1.22 | U= 1506.00 | 0.149 |
| Median (Min. – Max.) | 13 (12 – 15) | 14 (11 – 15) |  |  |
| **INR** |  |  |  |  |
| Mean ± SD. | 1.01 ± 0.07 | 0.99 ± 0.06 | U= 1750.500 | 0.801 |
| Median (Min. – Max.) | 1 (0.80 – 1.30) | 1 (0.80 – 1.02) |  |  |
| **IL-6 (pg/mL)** |  |  |  |  |
| Mean ± SD. | 167.6 ± 87.2 | 201.8 ± 69.5 | U= 1366.500^*^ | 0.041^*^ |
| Median (Min. – Max.) | 195 (20 – 350) | 215 (21 – 350) |  |  |

Hb: Hemoglobin concentration, WBCs: white blood cells, CRP: C reactive protein, ALT: Alanine transaminase, LDH: Lactate Dehydrogenase, PT: prothrombin time, INR: International normalized ratio, IL-6: Interleukin-6, SD: Standard deviation, U: Mann Whitney test, χ^2^: Chi square test, p: p value, *: Statistically significant at p < 0.05

**Supplementary table 2: Relation between outcome and clinical assessment**

|  | **Outcome** | | **χ^2^** | **p** |
| --- | --- | --- | --- | --- |
|  | **Alive (n = 120)** | **Died (n = 30)** |  |  |
| **Fever** | 96 (80%) | 30 (100%) | 7.143^*^ | ^FE^p=0.004^*^ |
| **Cough** | 108 (90%) | 30 (100%) | 3.261 | ^FE^p=0.125 |
| **Sputum** | 51 (42.5%) | 24 (80%) | 13.500^*^ | >0.001^*^ |
| **Dyspnea** | 78 (65%) | 30 (100%) | 14.583^*^ | >0.001^*^ |
| **Hemoptysis** | 1 (0.8%) | 2 (6.7%) | 4.167 | ^FE^p=0.102 |
| **Cyanosis** | 11 (9.2%) | 7 (23.3%) | 4.561 | ^FE^p=0.054 |
| **Myalgia** | 41 (34.2%) | 1 (3.3%) | 11.318^*^ | 0.001^*^ |
| **Bone ache** | 58 (48.3%) | 5 (16.7%) | 9.880^*^ | 0.002^*^ |
| **Anosmia** | 69 (57.5%) | 6 (20%) | 13.500^*^ | >0.001^*^ |
| **Loss of taste** | 37 (30.8%) | 5 (16.7%) | 2.389 | 0.122 |
| **Vomiting** | 26 (21.7%) | 1 (3.3%) | 5.465^*^ | 0.019^*^ |
| **Diarrhea** | 62 (51.7%) | 4 (13.3%) | 14.313^*^ | >0.001^*^ |
| **Conjunctivitis** | 39 (32.5%) | 15 (50%) | 3.190 | 0.074 |
| **Chills** | 10 (8.3%) | 8 (26.7%) | 7.639^*^ | ^FE^p=0.011^*^ |
| **Runny nose** | 18 (15%) | 0 (0%) | 5.114^*^ | ^FE^p=0.024^*^ |
| **CO-RADS** |  |  |  |  |
| 0 | 8 (6.7%) | 0 (0%) | 35.685^*^ | ^MC^p >0.001^*^ |
| 3 | 7 (5.8%) | 0 (0%) |  |  |
| 4 | 54 (45%) | 0 (0%) |  |  |
| 5 | 51 (42.5%) | 30 (100%) |  |  |
| **History of DM** | 48 (40%) | 27 (90%) | 24.000^*^ | >0.001^*^ |
| **History of HTN** | 45 (37.5%) | 24 (80%) | 17.452^*^ | >0.001^*^ |

DM: Diabetes mellitus, HTN: Hypertension, χ^2^: Chi square test, FE: Fisher Exact, MC: Monte Carlo, p: p value, *: Statistically significant at p < 0.05

**Supplementary table 3: Relation between *FURIN* genotypes, demographic and laboratory data in patients group**

|  | ***FURIN*** | | | **Test of Sig.** | **p** |
| --- | --- | --- | --- | --- | --- |
|  | **G/G (n = 40)** | **G/C (n = 63)** | **C/C (n = 47)** |  |  |
| **Sex** |  |  |  |  |  |
| Male | 25 (62.5%) | 30 (47.6%) | 29 (61.7%) | χ^2^= 3.102 | 0.212 |
| Female | 15 (37.5%) | 33 (52.4%) | 18 (38.3%) |  |  |
| **Age (years)** |  |  |  |  |  |
| Mean ± SD. | 55.4 ± 14.7 | 56.8 ± 17.2 | 59.7 ± 16.5 | H= 2.073 | 0.355 |
| Median (Min. – Max.) | 58.5 (33 – 86) | 53 (25 – 88) | 63 (25 – 88) |  |  |
| **HB** |  |  |  |  |  |
| Mean ± SD. | 11.3 ± 1.7 | 10.9 ± 1.6 | 10.9 ± 1.5 | H= 1.498 | 0.473 |
| Median (Min. – Max.) | 11 (7.5 – 14) | 11 (7.5 – 14) | 11 (8.5 – 14) |  |  |
| **WBCs** |  |  |  |  |  |
| Mean ± SD. | 8.1 ± 4.3 | 10.9 ± 7.1 | 8.6 ± 4.9 | H= 4.588 | 0.101 |
| Median (Min. – Max.) | 9 (3.8 – 28) | 9.5 (3.8 – 32) | 7 (3.8 – 25) |  |  |
| **Lymphocytes (%)** |  |  |  |  |  |
| Mean ± SD. | 13.8 ± 2.7 | 13.7 ± 4.7 | 14.4 ± 4.4 | H= 1.586 | 0.452 |
| Median (Min. – Max.) | 13 (7 – 19) | 13 (7 – 23) | 14 (5 – 22) |  |  |
| **Segmented (%)** |  |  |  |  |  |
| Mean ± SD. | 77.8 ± 7.8 | 80.8 ± 7.9 | 78.5 ± 8 | H= 6.200^*^ | 0.045^*^ |
| Median (Min. – Max.) | 78 (38 – 90) | 80 (38 – 90) | 80 (38 – 90) |  |  |
| **Sig. between groups** | **p_1_=0.018^*^, p_2_=0.481, p_3_=0.091** | | |  |  |
| **Platelet count** |  |  |  |  |  |
| Mean ± SD. | 242.9 ± 84.2 | 246.6 ± 78.6 | 272.7 ± 83.3 | H= 3.874 | 0.144 |
| Median (Min. – Max.) | 212.5 (129 – 430) | 215 (140 – 430) | 283 (154 – 450) |  |  |
| **CRP** |  |  |  |  |  |
| Mean ± SD. | 58 ± 50.4 | 84.4 ± 58.4 | 76 ± 48.1 | H= 7.700^*^ | 0.021^*^ |
| Median (Min. – Max.) | 48 (12 – 196) | 96 (12 – 196) | 48 (12 – 196) |  |  |
| **Sig. between groups** | **p_1_=0.009^*^, p_2_=0.023^*^, p_3_=0.851** | | |  |  |
| **Serum Creatinine** |  |  |  |  |  |
| Mean ± SD. | 1.04 ± 0.15 | 1.13 ± 0.33 | 1.08 ± 0.18 | H= 2.323 | 0.313 |
| Median (Min. – Max.) | 1 (0.80 – 1.50) | 1 (0.80 – 2.10) | 1.02 (0.80 – 1.60) |  |  |
| **Blood urea** |  |  |  |  |  |
| Mean ± SD. | 40.9 ± 16.2 | 48.5 ± 23 | 41.1 ± 7 | H= 4.187 | 0.123 |
| Median (Min. – Max.) | 36.5 (25 – 102) | 45 (25 – 102) | 45 (25 – 50) |  |  |
| **ALT** |  |  |  |  |  |
| Mean ± SD. | 36.5 ± 16.7 | 40 ± 23.4 | 52.9 ± 57.5 | H= 0.402 | 0.818 |
| Median (Min. – Max.) | 35 (18 – 80) | 35 (18 – 120) | 28 (20 – 250) |  |  |
| **Serum ferritin** |  |  |  |  |  |
| Mean ± SD. | 547.9 ± 349.1 | 762.7 ± 501.9 | 685.4 ± 270.7 | H= 7.836^*^ | 0.020^*^ |
| Median (Min. – Max.) | 400 (200 – 1500) | 600 (200 – 2000) | 700 (300 – 1200) |  |  |
| **Sig. between groups** | **p_1_=0.023^*^, p_2_=0.008^*^, p_3_=0.574** | | |  |  |
| **LDH** |  |  |  |  |  |
| Mean ± SD. | 524 ± 373.6 | 709.5 ± 430.9 | 674.3 ± 274.4 | H= 11.829^*^ | 0.003^*^ |
| Median (Min. – Max.) | 340 (280 – 1800) | 600 (280 – 1800) | 650 (280 – 1200) |  |  |
| **Sig. between groups** | **p_1_=0.005^*^, p_2_=0.001^*^, p_3_=0.487** | | |  |  |
| **D-Dimer** |  |  |  |  |  |
| Mean ± SD. | 0.77 ± 0.83 | 1.14 ± 0.94 | 1.02 ± 0.56 | H= 8.841^*^ | 0.012^*^ |
| Median (Min. – Max.) | 0.40 (0.10 – 3.50) | 1 (0.10 – 3.50) | 1 (0.10 – 2.50) |  |  |
| **Sig. between groups** | **p_1_=0.009^*^, p_2_=0.007^*^, p_3_=0.788** | | |  |  |
| **Prolcalcitonin** |  |  |  |  |  |
| Mean ± SD. | 0.26 ± 0.36 | 0.47 ± 0.49 | 0.48 ± 0.43 | H= 12.044^*^ | 0.002^*^ |
| Median (Min. – Max.) | 0.17 (0.01 – 1.50) | 0.25 (0.01 – 1.50) | 0.35 (0.01 – 1.50) |  |  |
| **Sig. between groups** | **p_1_=0.006^*^, p_2_=0.001^*^, p_3_=0.435** | | |  |  |
| **PT** |  |  |  |  |  |
| Mean ± SD. | 12.9 ± 0.9 | 13.1 ± 1.1 | 13.6 ± 1 | H= 10.708^*^ | 0.005^*^ |
| Median (Min. – Max.) | 12.5 (12 – 14) | 13 (11 – 15) | 14 (12 – 15) |  |  |
| **Sig.bet.Grps** | p_1_=0.240,p_2_=0.002^*^,p_3_=0.021^*^ | | |  |  |
| **INR** |  |  |  |  |  |
| Mean ± SD. | 0.98 ± 05 | 12 ± 08 | 10 ± 07 | H= 5.477 | 0.065 |
| Median (Min. – Max.) | 10 (0.80 – 12) | 10 (0.80 – 1.30) | 11 (0.80 – 1.20) |  |  |
| **IL-6** |  |  |  |  |  |
| Mean ± SD. | 144.4 ± 86.2 | 178.5 ± 82.6 | 194.7 ± 81.2 | H= 12.794^*^ | 0.002^*^ |
| Median (Min. – Max.) | 180 (20 – 350) | 205 (20 – 350) | 220 (20 – 320) |  |  |
| **Sig. between groups** | **p_1_=0.028^*^, p_2_<0.001^*^, p_3_=0.094** | | |  |  |

Hb: hemoglobin concentration, WBCs: white blood cells, CRP: C reactive protein, ALT: Alanine transaminase, LDH: Lactate Dehydrogenase, PT: prothrombin time, INR: International normalized ratio, IL-6: Interleukin-6, SD: Standard deviation, χ2: Chi square test, H: H for Kruskal Wallis test, Pairwise comparison between each 2 groups was done using Post Hoc Test (Dunn's for multiple comparisons test), p: p value, p1: p value for comparing between G/G and G/C, p_2_: p value for comparing between G/G and C/C, p_3_: p value for comparing between **G/C** and **C/C**, *: Statistically significant at p ≤ 0.05

**Supplementary table 4: Relation between *IFNL4* genotypes, demographic and laboratory data in patients group**

|  | ***IFNL4*** | | | **Test of Sig.** | **p** |
| --- | --- | --- | --- | --- | --- |
|  | **C/C (n = 54)** | **C/T (n = 62)** | **T/T (n = 34)** |  |  |
| **Sex** |  |  |  |  |  |
| Male | 25 (46.3%) | 39 (62.9%) | 20 (58.8%) | χ^2^= 3.373 | 0.185 |
| Female | 29 (53.7%) | 23 (37.1%) | 14 (41.2%) |  |  |
| **Age (years)** |  |  |  |  |  |
| Mean ± SD. | 54.5 ± 16.7 | 58.3 ± 16.3 | 60 ± 15.6 | H= 2.914 | 0.233 |
| Median (Min. – Max.) | 52.5 (25 – 85) | 62 (25 – 86) | 56 (36 – 88) |  |  |
| **HB** |  |  |  |  |  |
| Mean ± SD. | 11.2 ± 1.6 | 11 ± 1.6 | 10.8 ± 1.6 | H= 1.860 | 0.395 |
| Median (Min. – Max.) | 11 (8.5 – 14) | 11 (7.5 – 14) | 11 (8.5 – 14) |  |  |
| **WBCs** |  |  |  |  |  |
| Mean ± SD. | 9.3 ± 4.8 | 9.3 ± 5.8 | 9.8 ± 7.6 | H= 0.584 | 0.747 |
| Median (Min. – Max.) | 9.5 (3.8 – 25) | 9.8 (3.8 – 28) | 8.7 (3.9 – 32) |  |  |
| **Lymphocytes (%)** |  |  |  |  |  |
| Mean ± SD. | 13.7 ± 4.3 | 14 ± 3.9 | 14.3 ± 4.3 | H= 0.488 | 0.784 |
| Median (Min. – Max.) | 13 (5 – 22) | 14 (5 – 23) | 13.5 (7 – 20) |  |  |
| **Segmented (%)** |  |  |  |  |  |
| Mean ± SD. | 79.6 ± 9.6 | 78.3 ± 7.6 | 80.5 ± 5.2 | H= 3.765 | 0.152 |
| Median (Min. – Max.) | 80 (38 – 90) | 78 (38 – 90) | 80 (72 – 90) |  |  |
| **Platelet count** |  |  |  |  |  |
| Mean ± SD. | 260.6 ± 82.9 | 258.1 ± 76.4 | 235.1 ± 90.2 | H= 3.742 | 0.154 |
| Median (Min. – Max.) | 245 (129 – 430) | 235 (145 – 430) | 195 (129 – 450) |  |  |
| **CRP** |  |  |  |  |  |
| Mean ± SD. | 70.6 ± 55.4 | 72.5 ± 53.2 | 85.5 ± 53.3 | H= 4.071 | 0.131 |
| Median (Min. – Max.) | 48 (12 – 196) | 48 (12 – 196) | 96 (12 – 196) |  |  |
| **Serum Creatinine** |  |  |  |  |  |
| Mean ± SD. | 1.1 ± 0.2 | 1.1 ± 0.2 | 1.2 ± 0.3 | H= 1.645 | 0.439 |
| Median (Min. – Max.) | 1 (0.8 – 1.9) | 1 (0.8 – 1.9) | 1 (0.8 – 2.1) |  |  |
| **Blood urea** |  |  |  |  |  |
| Mean ± SD. | 41.1 ± 12.3 | 43.2 ± 16.8 | 50.6 ± 24.7 | H= 1.557 | 0.459 |
| Median (Min. – Max.) | 40 (25 – 86) | 40 (25 – 102) | 45 (25 – 100) |  |  |
| **ALT** |  |  |  |  |  |
| Mean ± SD. | 34.2 ± 16.7 | 38.6 ± 21.5 | 65.6 ± 64.7 | H= 10.823^*^ | 0.004^*^ |
| Median (Min. – Max.) | 28 (18 – 110) | 35 (18 – 110) | 45 (18 – 250) |  |  |
| **Sig. between groups** | **p_1_=0.550, p_2_=0.002^*^, p_3_=0.007^*^** | | |  | |
| **Serum ferritin** |  |  |  |  |  |
| Mean ± SD. | 642.8 ± 426.4 | 680.9 ± 411.4 | 742.8 ± 377.6 | H= 2.891 | 0.236 |
| Median (Min. – Max.) | 500 (300 – 2000) | 500 (300 – 2000) | 700 (200 – 1500) |  |  |
| **LDH** |  |  |  |  |  |
| Mean ± SD. | 590.2 ± 355.9 | 706.6 ± 448.3 | 637.4 ± 242.2 | H= 3.518 | 0.172 |
| Median (Min. – Max.) | 425 (280 – 1500) | 600 (280 – 1800) | 600 (290 – 1000) |  |  |
| **D-Dimer** |  |  |  |  |  |
| Mean ± SD. | 0.91 ± 0.86 | 0.95 ± 0.75 | 1.26 ± 0.83 | H= 7.268^*^ | 0.026^*^ |
| Median (Min. – Max.) | 0.65 (0.10 – 3.50) | 0.95 (0.10 – 3.50) | 1.00 (0.28 – 3.50) |  |  |
| **Sig. between groups** | **p_1_=0.394, p_2_=0.008^*^, p_3_=0.046^*^** | | |  |  |
| **Prolcalcitonin** |  |  |  |  |  |
| Mean ± SD. | 0.32 ± 0.35 | 0.39 ± 0.45 | 0.61 ± 0.52 | H= 13.870^*^ | 0.001^*^ |
| Median (Min. – Max.) | 0.20 (0.01 – 1.50) | 0.20 (0.01 – 1.50) | 0.40 (0.01 – 1.50) |  |  |
| **Sig. between groups** | **p_1_=0.395, p_2_=<0.001^*^, p_3_=0.003^*^** | | |  |  |
| **PT** |  |  |  |  |  |
| Mean ± SD. | 12.9 ± 1.0 | 13.2 ± 1.1 | 13.5 ± 1.1 | H= 6.955^*^ | 0.031^*^ |
| Median (Min. – Max.) | 13.0 (11.0 – 15.0) | 13.0 (11.0 – 15.0) | 14.0 (12.0 – 15.0) |  |  |
| **Sig. between groups** | **p_1_=0.077, p_2_=0.011^*^, p_3_=0.285** | | |  |  |
| **INR** |  |  |  |  |  |
| Mean ± SD. | 1 ± 0.07 | 1 ± 0.05 | 1.01 ± 0.11 | H= 0.153 | 0.927 |
| Median (Min. – Max.) | 1 (0.8 – 1.2) | 1 (0.8 – 1.2) | 1 (0.8 – 1.3) |  |  |
| **IL-6** |  |  |  |  |  |
| Mean ± SD. | 181.8 ± 84.7 | 143.2 ± 82.9 | 219.9 ± 64.9 | H= 16.968^*^ | <0.001^*^ |
| Median (Min. – Max.) | 205 (20 – 320) | 180 (20 – 255) | 222.5 (50 – 350) |  |  |
| **Sig. between groups** | **p_1_=0.023^*^, p_2_<0.001^*^, p_3_=0.043^*^** | | |  |  |

Hb: hemoglobin concentration, WBCs: white blood cells, CRP: C reactive protein, ALT: Alanine transaminase, LDH: Lactate Dehydrogenase, PT: prothrombin time, INR: International normalized ratio, IL-6: Interleukin-6, SD: Standard deviation, χ2: Chi square test, H: H for Kruskal Wallis test, Pairwise comparison between each 2 groups was done using Post Hoc Test (Dunn's for multiple comparisons test), p: p value, p1: p value for comparing between C/C and C/T, p_2_: p value for comparing between C/C and T/T, p_3_: p value for comparing between C/T and T/T, *: Statistically significant at p ≤ 0.05

**Supplementary table 5: Relation between *TLR2* genotypes, demographic and laboratory data in patients group**

|  | ***TLR2*** | | | **Test of Sig.** | **p** |
| --- | --- | --- | --- | --- | --- |
|  | **T/T (n = 35)** | **T/C (n = 64)** | **C/C (n = 51)** |  |  |
| **Sex** |  |  |  |  |  |
| Male | 22 (62.9%) | 37 (57.8%) | 25 (49%) | χ^2^= 1.762 | 0.414 |
| Female | 13 (37.1%) | 27 (42.2%) | 26 (51%) |  |  |
| **Age (years)** |  |  |  |  |  |
| Mean ± SD. | 51.9 ± 16.9 | 56.9 ± 16.1 | 61.5 ± 15.3 | H= 7.167^*^ | 0.028^*^ |
| Median (Min. – Max.) | 50 (25 – 88) | 60 (25 – 86) | 60 (35 – 88) |  |  |
| **Sig. between groups** | **p_1_=0.141, p_2_=0.008^*^, p_3_=0.141** | | |  |  |
| **HB** |  |  |  |  |  |
| Mean ± SD. | 10.9 ± 1.4 | 11 ± 1.8 | 11.1 ± 1.4 | H= 0.671 | 0.715 |
| Median (Min. – Max.) | 11 (8.5 – 13) | 11 (7.5 – 14) | 11 (7.5 – 14) |  |  |
| **WBCs** |  |  |  |  |  |
| Mean ± SD. | 7.7 ± 3.3 | 10.7 ± 7.5 | 9.1 ± 4.5 | H= 2.865 | 0.239 |
| Median (Min. – Max.) | 8 (3.8 – 15) | 10 (3.8 – 32) | 9 (3.8 – 28) |  |  |
| **Lymphocytes (%)** |  |  |  |  |  |
| Mean ± SD. | 14.8 ± 3.6 | 13.7 ± 3.9 | 13.6 ± 4.8 | H= 2.087 | 0.352 |
| Median (Min. – Max.) | 15 (7 – 23) | 14 (5 – 20) | 13 (5 – 23) |  |  |
| **Semented (%)** |  |  |  |  |  |
| Mean ± SD. | 78.8 ± 4.6 | 78.8 ± 9.2 | 80.3 ± 8.2 | H= 2.797 | 0.247 |
| Median (Min. – Max.) | 78 (70 – 90) | 80 (38 – 90) | 80 (38 – 90) |  |  |
| **PLT** |  |  |  |  |  |
| Mean ± SD. | 270.2 ± 79 | 254.4 ± 85.6 | 241.8 ± 79.2 | H= 4.154 | 0.125 |
| Median (Min. – Max.) | 240 (129 – 450) | 235 (129 – 450) | 200 (129 – 450) |  |  |
| **CRP** |  |  |  |  |  |
| Mean ± SD. | 66.7 ± 46.8 | 77.5 ± 60.2 | 76.8 ± 50.7 | H= 1.165 | 0.559 |
| Median (Min. – Max.) | 48 (12 – 196) | 48 (12 – 196) | 95 (12 – 196) |  |  |
| **Serum Creatinine** |  |  |  |  |  |
| Mean ± SD. | 1.04 ± 0.23 | 1.06 ± 0.15 | 1.15 ± 0.34 | H= 2.643 | 0.267 |
| Median (Min. – Max.) | 1.0 (0.80 – 2.10) | 1.0 (0.80 – 1.50) | 1.0 (0.80 – 2.10) |  |  |
| **Blood urea** |  |  |  |  |  |
| Mean ± SD. | 41.5 ± 14.6 | 44.2 ± 18.2 | 45.9 ± 19.3 | H= 1.418 | 0.492 |
| Median (Min. – Max.) | 38 (25 – 100) | 40 (25 – 102) | 45 (25 – 102) |  |  |
| **ALT** |  |  |  |  |  |
| Mean ± SD. | 49.2 ± 43.9 | 40 ± 33.4 | 42.8 ± 36.4 | H= 1.501 | 0.472 |
| Median (Min. – Max.) | 35 (18 – 250) | 32.5 (18 – 250) | 35 (18 – 250) |  |  |
| **Serum ferritin** |  |  |  |  |  |
| Mean ± SD. | 607.3 ± 328.2 | 643.9 ± 356.4 | 778.7 ± 498.9 | H= 2.921 | 0.232 |
| Median (Min. – Max.) | 500 (300 – 1500) | 500 (200 – 1500) | 600 (200 – 2000) |  |  |
| **LDH** |  |  |  |  |  |
| Mean ± SD. | 551.7 ± 289.4 | 631.4 ± 392.7 | 737.8 ± 399.7 | H= 5.712 | 0.058 |
| Median (Min. – Max.) | 400 (280 – 1200) | 550 (280 – 1800) | 600 (280 – 1800) |  |  |
| **D-Dimer** |  |  |  |  |  |
| Mean ± SD. | 0.82 ± 0.79 | 0.90 ± 0.72 | 1.26 ± 0.89 | H= 10.438^*^ | 0.005^*^ |
| Median (Min. – Max.) | 0.50 (0.10 – 3.50) | 0.80 (0.10 – 3.50) | 1.0 (0.28 – 3.50) |  |  |
| **Sig. between groups** | **p_1_=0.438, p_2_=0.003^*^, p_3_=0.010^*^** | | |  |  |
| **Prolcalcitonin** |  |  |  |  |  |
| Mean ± SD. | 0.37 ± 0.46 | 0.40 ± 0.46 | 0.47 ± 0.43 | H= 5.515 | 0.063 |
| Median (Min. – Max.) | 0.18 (0.01 – 1.50) | 0.19 (0.01 – 1.50) | 0.35 (0.01 – 1.50) |  |  |
| **PT** |  |  |  |  |  |
| Mean ± SD. | 13 ± 1 | 13.3 ± 1 | 13.2 ± 1.1 | H= 1.693 | 0.429 |
| Median (Min. – Max.) | 13 (12 – 15) | 14 (11 – 15) | 13 (11 – 15) |  |  |
| **INR** |  |  |  |  |  |
| Mean ± SD. | 1.01 ± 0.06 | 1 ± 0.06 | 1 ± 0.09 | H= 1.492 | 0.474 |
| Median (Min. – Max.) | 1 (0.90 – 1.30) | 1 (0.80 – 1.20) | 1 (0.80 – 1.30) |  |  |
| **IL-6** |  |  |  |  |  |
| Mean ± SD. | 175.8 ± 77.5 | 160.4 ± 86.7 | 191.2 ± 85.8 | H= 6.124^*^ | 0.047^*^ |
| Median (Min. – Max.) | 205 (35 – 320) | 187.5 (20 – 350) | 210 (20 – 350) |  |  |
| **Sig. between groups** | **p_1_=0.229, p_2_=0.340, p_3_=0.014^*^** | | |  |  |

Hb: hemoglobin concentration, WBCs: white blood cells, CRP: C reactive protein, ALT: Alanine transaminase, LDH: Lactate Dehydrogenase, PT: prothrombin time, INR: International normalized ratio, IL-6: Interleukin-6, SD: Standard deviation, χ2: Chi square test, H: H for Kruskal Wallis test, Pairwise comparison between each 2 groups was done using Post Hoc Test (Dunn's for multiple comparisons test), p: p value, p1: p value for comparing between T/T and T/C, p_2_: p value for comparing between T/T and T/C, p_3_: p value for comparing between T/C and C/C, *: Statistically significant at p ≤ 0.05
